# A Structural and *In Silico* Investigation of Potential CDC7 Kinase Enzyme Inhibitors

**DOI:** 10.1021/acsomega.3c07059

**Published:** 2023-11-27

**Authors:** Mohanbabu Mookkan, Saravanan Kandasamy, Abdel-Basit Al-Odayni, Naaser Ahmed Yaseen Abduh, Sugarthi Srinivasan, Bistuvalli Chandrashekara Revannasidappa, Vasantha Kumar, Kalaiarasi Chinnasamy, Sanmargam Aravindhan, Madan Kumar Shankar

**Affiliations:** † Department of Physics, Presidency College (Autonomous), University of Madras, Chennai 600 005, India; ‡ Faculty of Chemistry, University of Warsaw, Ludwika Pasteura 1, Warsaw 02-093, Poland; § Department of Restorative Dental Science, College of Dentistry, King Saud University, P.O. Box 60169, Riyadh 11545, Saudi Arabia; ∥ Department of Chemistry, College of Science, King Saud University, P.O. Box 2455, Riyadh 11451, Saudi Arabia; ⊥ Department of Physics and Nanotechnology, SRM Institute of Science and Technology, Kattankulathur 603203, India; # Department of Pharmaceutical Chemistry, NGSM Institute of Pharmaceutical Sciences of Nitte - Deemed to be University, Paneer, Deralakatte, Mangalore 575018, Karnataka India; ∇ Department of P.G. Chemistry, Sri Dharmasthala Manjunatheshwara College (Autonomous), Ujire 574240, India; ○ Molecular Biophysics Unit, Indian Institute of Science, Bangalore 560 012, India; ◆ Department of Chemistry-BMC, University of Uppsala, Husargatan 3, Uppsala 75237, Sweden

## Abstract

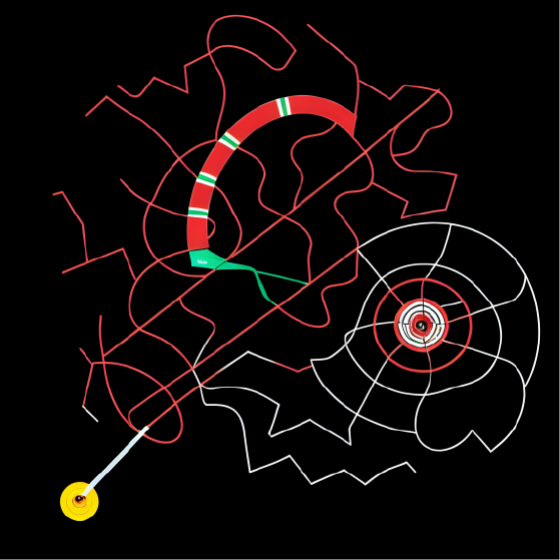

A crucial role in the regulation of DNA replication is played by the highly conserved CDC kinase. The CDC7 kinase could serve as a target for therapeutic intervention in cancer. The primary heterocyclic substance is pyrazole, and its derivatives offer great potential as treatments for cancer cell lines. Here, we synthesized the two pyrazole derivatives: 4-(2-(4-chlorophenyl)hydrazinyl)-5-methyl-2-tosyl-1H-pyrazol-3(2H)-one (PYRA-1) and 4-(2-(2,4-difluorophenyl)hydrazinyl)-5-methyl-2-tosyl-1H-pyrazol-3(2H)-one (PYRA-2). The structural confirmation of both the compounds at the three-dimensional level is characterized using single crystal X-ray diffraction and density functional theory. Furthermore, the *in silico* chemical biological properties were derived using molecular docking and molecular dynamics (MD) simulations. PYRA-1 and PYRA-2 crystallize in the P-1 (*a* = 8.184(9), *b* = 14.251(13), *c* = 15.601(15), α = 91.57(8), β = 97.48(9), 92.67(9), *V* = 1801.1(3) 3, and *Z* = 2) and P2_1_/*n* (*a* = 14.8648(8), *b* = 8.5998(4), *c* = 15.5586(8), β = 116.47(7), *V* = 1780.4(19) 3, and *Z* = 4), space groups, respectively. In both PYRA-1 and PYRA-2 compounds, C–H···O intermolecular connections are common to stabilize the crystal structure. In addition, short intermolecular interactions stabilizes with C–H···π and π–π stacking. Crystal packing analysis was quantified using Hirshfeld surface analysis resulting in C···H, O···H, and H···H contacts in PYRA-1 exhibiting more contribution than in PYRA-2. The conformational stabilities of the molecules are same in the gas and liquid phases (water and DMSO). The docking scores measured for PYRA-1 and PYRA-2 with CDC7 kinase complexes are −5.421 and −5.884 kcal/mol, respectively. The MD simulations show that PYRA-2 is a more potential inhibitor than PYRA-1 against CDC7 kinase.

## Introduction

Pyrazole and its derivatives are the fundamental heterocyclic compound in the aromatic organics society and have tremendous potential against cancer cell lines (prostate, skin, leukemia, breast, ovarian, and kidney cancers).^1^ Pyrazoles have been utilized in wide-ranging pharmaceutic applications, such as antimalarial,^2,3^ anti-inflammatory,^4^ antimicrobial,^5^*in vitro* antifungal activity, and antitumor agents in medicinal therapy.^6^ They possess extensive potential in biological applications, such as antihistaminic,^7^ antidepressant,^8^ antifungal activities,^9^ and in the treatment of anxiety.^10^ Pyrazole derivatives have been used as antiparasitic agents against *Trichomonas vaginalis* and have antipyretic activity.^11^ They have spectacle analogs in pharmaceutical drugs, such as *Rimonabant* and *Celebrex*.^12^ Especially they have acted as corrosion inhibitors of mild steel.^13^ In agrochemical industries, their derivatives have also been used as insecticides and as an intermediate in pesticides and herbicides. 1H-Pyrazole derivatives with phenyl groups have been prescribed for antibacterial activity against Gram-positive bacteria, such as *Bacillus subtilis*, *Staphylococcus*, and Gram-negative bacteria, such as *Escherichia coli*, *Pseudomonas aeruginosa* and antifungal activity against *Candida albicans* and *Aspergillus niger* at various concentrations (25, 50, 75, 100 μg/mL).^14^ Notably, they exhibit fluorescence, photoluminescence in blue, and electroluminescence characteristics.^15^ It was also reported that *in vivo* LQFM021 has an antinociceptive effect on receptors with NO/cGMP/K_ATP_ activation^16^ and possesses cardiovascular potential.^17^

Most of the malignant tumors with generative variants frequently exhibit dysfunctional responses, which enable tumor cells to continue to exist and transmit; this is an advantage in survival when the genome is copied. A lot of research has already been conducted to clarify the biological processes that control the regulation of cell cycles and the manner in which these systems are compromised in the development of tumors. Numerous human cancers are caused by abnormalities in proteins. With better knowledge of these functions, new targets for cancer treatment are being revealed. Among them, CDC7 (cell division cycle 7) is a serine/threonine kinase that is essential for the regular progression during the cell cycle. And, it also involves multiple links between its structure and function with the CDKs. It seems to be an essential target of therapeutic control. A lot is being discovered concerning the biological functions of CDC7 kinase in humans via studies involving eukaryotes, especially yeasts. This enzyme has undergone significant levels of conservation throughout evolution.^18,19^ Based on reports,^20−26^ there are many organic compounds and their derivatives that show CDC7 kinase activity. The causes for these applications of pyrazole and its derivatives in medicinal and biological fields, which we report in this paper, are the synthesis, structural investigation, Hirshfeld surface interactions, DFT computation theory, bioinformatics studies, and molecular stability of the pyrazole compounds.

## Methods

### Synthesis and Crystallization

#### PYRA-1

**Scheme 1:**
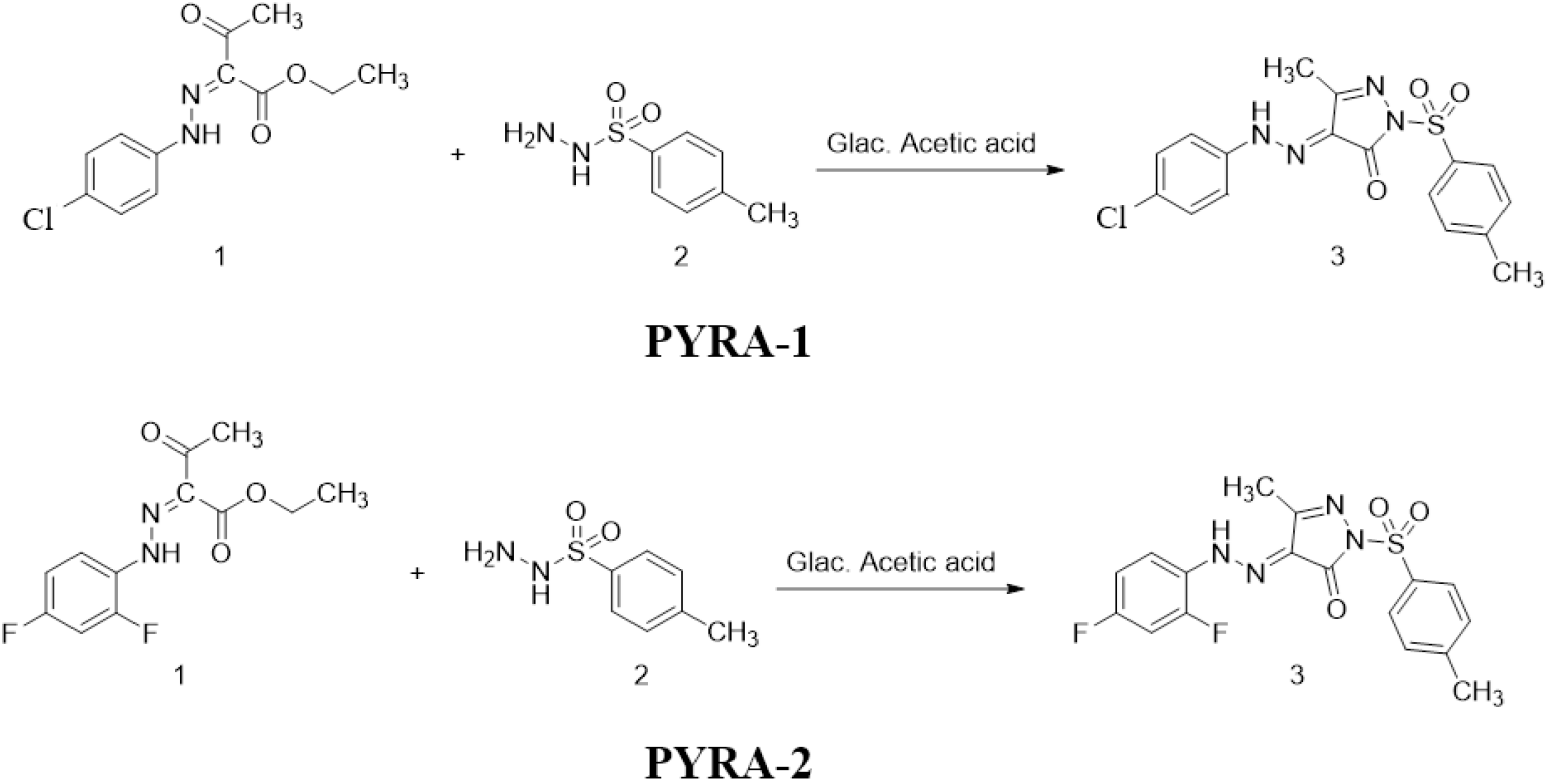
Synthetic Route of PYRA-1 and PYRA-2

Ethyl(*E*)-2-(2-(2,4-chlorophenyl)hydrazineylidene)-3-oxobutanoate (**1**) (0.01 mol) was dissolved in 25 mL of glacial acetic acid. To this, a solution of *p*-toluene sulphonyl carbohydrazide (**2**) (0.01 mol) in glacial acetic acid was added, and the reaction mixture was refluxed for about 24–34 h. After cooling, the reaction mixture was poured into ice water and stirred. The solid precipitate was filtered, washed with water, and recrystallized from the ethanol–DMF mixture (Scheme 1). The melting point, FT-IR, and ^1^H NMR graphs are shown in Figure S1.

#### PYRA-2

Ethyl(*E*)-2-(2-(2,4-difluorophenyl)hydrazineylidene)-3-oxobutanoate (**1**) (0.01 mol) was dissolved in 25 mL of glacial acetic acid. To this, a solution of *p*-toluene sulphonyl carbohydrazide (**2**) (0.01 mol) in glacial acetic acid was added, and the reaction mixture was refluxed for about 24–34 h. After cooling, the reaction mixture was poured into ice water and stirred. The solid precipitate was filtered, washed with water, and recrystallized from ethanol–DMF mixture (Scheme 1). The melting point and FT-IR graph are shown in Figure S1.

### X-ray Crystallography

X-ray diffraction intensity data were collected for PYRA-1 and PYRA-2 using a Rigaku diffractometer equipped with graphite monochromated Mo Kα (λ = 0.7103) radiation and a CCD detector. The data processing was carried out using Crystal Clear SM-Expert software.^27^ The unique reflections were used to solve the structures by direct methods, which are available in *SHELXS*.^28^ Furthermore, the structures were refined by the full-matrix least-squares method *SHELXL*.^29^ All the nonhydrogen atoms were first refined isotropically and then with anisotropic displacement parameters. All H atoms were positioned geometrically (C–H = 0.93–0.97) and allowed to ride on their parent C atoms with U_iso_(H) = 1.2–1.5UÅ(C) for methyl H atoms and 1.2U_eq_(C) for other H atoms. The geometrical measurements were calculated using *PLATON*(30), and the *MERCURY*(31) program was used for plotting Oak ridge thermal ellipsoid plot (ORTEP) and packing diagrams. The data collection and structure refinement statistics are listed in Table 1.

**Table 1:** Crystal Data and Structure Refinement for PYRA-1 and PYRA-2 Compounds

parameters	PYRA-1	PYRA-2
empirical formula	C_17_ H_15_ Cl N_4_ O_3_ S	C_17_ H_14_ F_2_ N_4_ O_3_ S
formula weight	780.79	392.38
temperature	293 K	293 K
wavelength	0.71073 Å	0.71073 Å
crystal system	triclinic	monoclinic
space group	P-1	*P*2_1_/*n*
unit cell dimensions	a = 8.184(9) α = 91.575(8)	a = 14.8648(8) α = 90
(a,b,c) Å	b = 14.251(13)β = 97.479(9)	b = 8.5998(4) β = 116.47(7)
(α, β, γ) °	c = 15.601(15) γ = 92.673(9)	c = 15.5586(8) γ = 90
volume	1801.1(3) Å^3^	1780.4(19) Å^3^
*Z*	2	4
density (calculated)	1.440 Mg/m^3^	1.464 Mg/m^3^
absorption coefficient	0.351 mm^-1^	0.228 mm^-1^
*F*(000)	807	808
crystal size	0.53 × 0.52 × 0.49 mm^3^	0.52 × 0.51 × 0.48 mm^3^
theta range for data collection	1.9–25.0°	2.8–26.4°
index ranges	–9 ≤ *h* ≤ 7, –16 ≤ *k* ≤ 16, –18 ≤ *l* ≤ 18	–18 ≤ *h* ≤ 18, –10 ≤ *k* ≤ 8, –19 ≤ *l* ≤ 19
reflections collected	17 517	19 241
independent reflections	6341 [*R*(int) = 0.0629]	3612 [*R*(int) = 0.0416]
completeness to theta = 25.242°	99.7%	99.9%
data/restraints/parameters	6341/0/475	3612/0/257
goodness-of-fit on *F*^2^	1.037	1.025
final *R* indices [*I* > 2sigma(I)]	R1 = 0.1381, wR2 = 0.3339	R1 = 0.0415, wR2 = 0.0988
*R* indices (all data)	R1 = 0.1683, wR2 = 0.3532	R1 = 0.0521, wR2 = 0.1071
Largest diff. peak and hole	0.82 and −0.34 eÅ^–3^	0.23 and −0.28 eÅ^–3^

### Hirshfeld Surface Analysis

Hirshfeld surface analysis was carried out using *Crystal Explorer 21.5* software,^32,33^ enabling the identification of the tight interactions between molecules in the crystal phase, allowing one to better understand the molecular packing in the crystal phase. The surface coverage ranging from red to blue between the internal *d*_i_ and external *d*_e_ surfaces was employed to construct Hirshfeld surfaces with a normalized contact distance (*d*_norm_). The shapes of fingerprint plots were used to describe the various intermolecular interactions of the molecules in the crystal as well as to assist in quantifying the contact distances to Hirshfeld surface analysis (*d*_i_, *d*_e_), which revealed the range from 0.6 to 2.8 considering reciprocal contacts.^34−40^ In order to comprehend the predicted structure–property relationships of the synthesized molecules, intermolecular interaction energies were determined at the B3LYP/6-31G(d,p) level of theory and categorized into electrostatic (*E*_elec_), polarization (*E*_pol_), dispersion (*E*_disp_), and repulsion (*E*_rep_) energy components.^35,39,40^

### DFT, QTAIM, and NCI Analysis

The molecules were optimized utilizing crystallographic coordinates using the B3LYP function of the Density Functional Theory (DFT) approach using the basis set 6-311G** using GAUSSIAN09 software^41^ to understand the gas phase geometry of the molecules. The energy minima of the molecules were reached using this comprehensive geometry optimization of quantum chemical calculations. The WinXPRO^42^ software’s 3Dplot was used to create the maps of electrostatic potential. Gaussview^43^ generated the highest occupied molecular orbital (HOMO) and lowest unoccupied molecular orbital (LUMO) maps. Using the Bader’s theory of AIM as implemented in MoPro software,^44^ the atom coordinates were used to derive the topological properties of interactions. In order to learn more about weak intermolecular interactions, these are helpful. Additionally, VMoPro^45−50^ software was used to create the NCI isosurface map for interactions discovered in the crystal phase and protein environment.

### Molecular Docking

The biological activity of both the pyrazole derivatives was predicted using the SWISS Target prediction system,^51^ which is based on the 2D and 3D of known existing drugs as well as inhibitors. According to the server’s prediction, these pyrazole compounds have kinase inhibitory and cancer-fighting properties. As a result, the protein data bank’s (PDB) 3D coordinates for CDC7 kinase (PDB ID: 4F9C) were obtained.^52^ In the following step, OPLS_2005 force field^53^ used the protein preparation wizard to prepare and minimize the protein. *Ligprep* was also used to optimize a few chosen ligands at the same time. To encompass every active site remnant listed on the sitemap, the grid was established at a 20 Å distance. The default scaling factor for the van der Waals’ radii of the atoms in the nonpolar receptor and ligand is 0.50. Last but not the least, the Glide and Prime modules included in the maestro of *Schrodinger* software^54^ were used to perform induced-fit docking (IFD) to accurately anticipate the ligand-binding mode and concurrent structural motions in the receptor. The optimal structural pose was then chosen from a set of 10 computed conformations based on the docking score, glide energy, hydrogen bonding, and hydrophobic bonding interactions.

### Molecular Dynamic Simulations

To further understand the stability of the complex in a time-constrained dynamic context, molecular dynamics (MD) simulations using Amber20 with the AMBER ff14SB force field^55^ were performed on the pose with the highest score achieved from docking investigations. For a duration of 100 ns, the MD simulation of the CDC7 complex was run. The solvated boundary for the prepared complex over 10 was constructed using TIP3P water models. In order to stabilize the complicated system’s charges, chlorine ions (Cl^–^) were also added. The SHAKE algorithm^56^ was used to limit the link lengths and angles within the protein during the simulation. To eliminate van der Waals short interactions, the MD protocol first subjected all hydrogen atoms, ions, and water molecules to 10 000 steps of energy minimization by steepest descent. The system goes through two equilibration phases in 500 ps. The first phase of an NVT ensemble is in which the thermostat exchanges the exothermic and endothermic processes. The NPT ensemble was maintained at 300 K with steady pressure in the second phase. At each phase, the velocities were assigned new values in accordance with the Maxwell–Boltzmann distribution as the temperature was raised to 300 K. The particle mesh Ewald method was used to analyze the electrostatic interactions between nonligand atoms using a 0.30 charge grid space. A 9.0 Å atom-based cutoff was used to assess the Lennard–Jones interactions.^57^ On a high-performance cluster computer, the MD simulation and result analysis were carried out. In terms of the root mean square deviation (RMSD) and the evolution of the hydrogen bonding from the initial structure, the convergence of the simulation was examined.

## Results and Discussions

### Structural Investigation

#### PYRA-1

**Figure 1:**
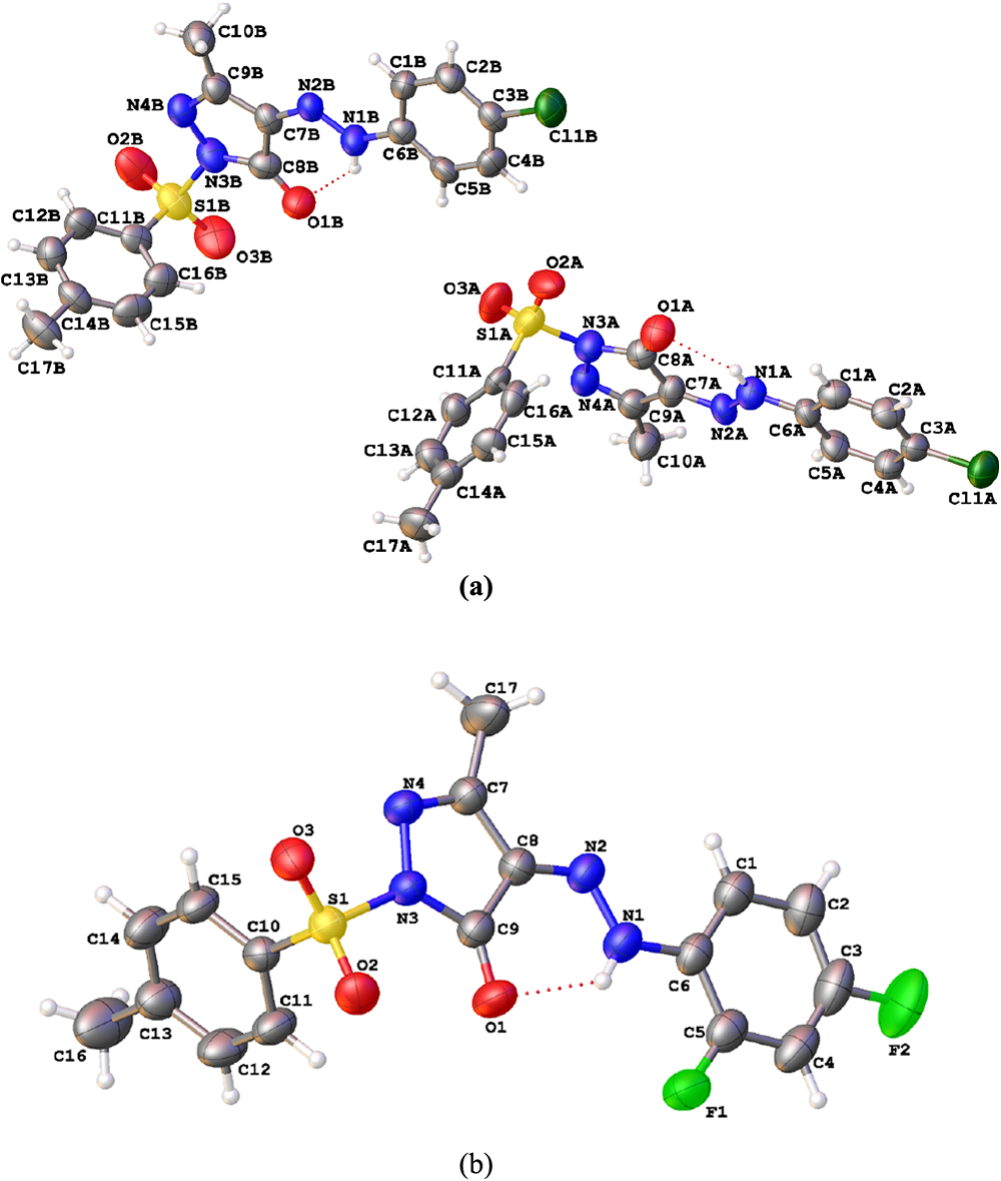
Thermal ellipsoid (50%) ORTEP view of (a) PYRA-1 and (b) PYRA-2 compounds.

**Figure 2:**
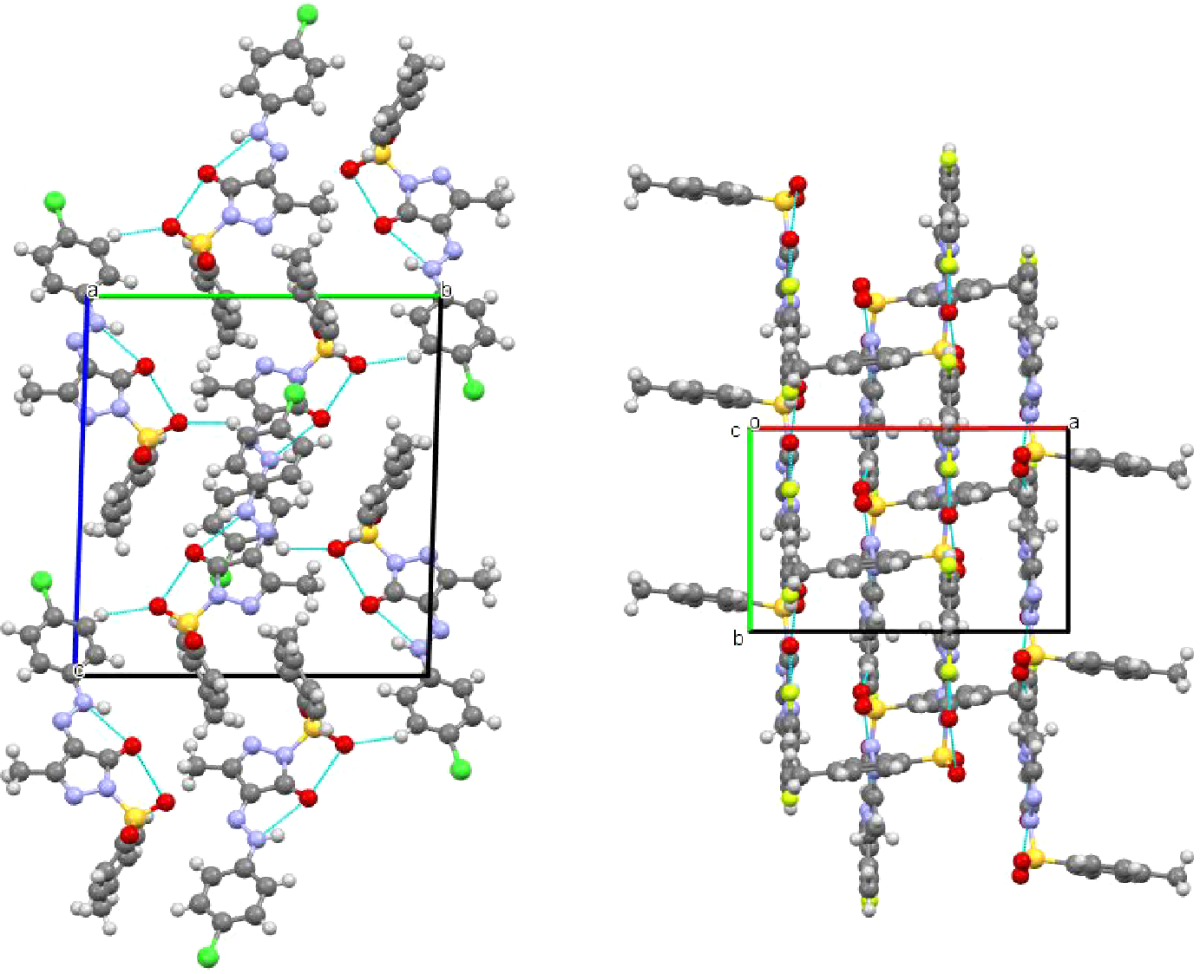
Packing along *a*-axis (left: PYRA-1) and along the *c*-axis (right: PYRA-2).

In this crystal structure, two asymmetric fragments (C_17_H_15_Cl N_4_O_3_ S) 4-(2-(4-chlorophenyl)hydrazinyl)-5-methyl-2-tosyl-1H-pyrazol-3(2H)-one (PYRA-1A and 1B) crystallized in a triclinic lattice with a space group *P-1* (Figure 1a). The dihedral angles between 4H-pyrazole and a chlorophenyl ring are 3.1(5)° (Mol-A) and 5.6(5)° (Mol-B). Intramolecular hydrogen bonds N1A···H1A···O1A, N1B···H1B···O1B, C12A···H12A···O3A, and C12B···H12B···O2A are observed (Table 2). The molecules are connected through the C2A–H2A···O3B, C4B–H4B···O2A, C15A–H15A···O3A, and C15B–H15B···O2A intermolecular hydrogen bonds (Table 2 and Figure 2). In addition, short intermolecular interactions of the type Cg1···Cg2 (3.603 (5)), Cg4···Cg5 (3.594(5) Å), C17···H17C···Cg3 (H···Cg = 2.91 ; 2-*x*, -*y*, 1-*z*; *X*···Cg = 3.705 (13); *X*···H···Cg = 141°), C3A···Cl1A···Cg1 (*X*···Cg = 3.968(5) ; 1-*x*, -*y*, -*z*; *Y*···Cg = 3.443 (10); *X*···H···Cg = 59.9 (3) °), and C3B···Cl1B···Cg4 (*X*···Cg = 3.983(5) ; -*x*, 1-*y*, 1-*z*; *Y*···Cg = 3.443 (10); *X*···H···Cg = 59.2(3) °), where Cg1: N3A–N4A–C9A–C7A–C8A, Cg2: C1A–C6A, Cg3: C11A–C16A, Cg4:N3B–N4B–C9B–C7B–C8B, Cg5: C1B–C6B, and Cg6:C11B–C16B.

**Table 2:** Hydrogen Bonding Interactions in the Crystal of PYRA-1 and PYRA-2 (Å, °)

symmetry codes: (i) 2–x,1–y,1–z; (ii) 1–x,1–y,1–z; (iii) 1+x,y,z; (iv) −1+x,y,z				
D–H···A	D–H	H···A	D···A	D–H···A
N1A–H1A···O1A	0.86	2.06	2.743(10)	136
N1B–H1B···O1B	0.86	2.08	2.765(9)	136
C2A–H2A**···**O3B^(i)^	0.93	2.36	3.172(11)	146
C4B–H4B···O2A^(ii)^	0.93	2.45	3.246(12)	143
C12A–H12A···O3A^(i)^	0.93	2.58	2.929(12)	102
C12B–H12B···O2B	0.93	2.57	2.919(12)	103
C15A–H15A···O3A^(iii)^	0.93	2.57	3.439(12)	155
C15B–H15B···O2B^(iv)^	0.93	2.54	3.403(13)	154

symmetry codes: (i) x,–1+y,z				
D–H···A	D–H	H···A	D···A	D–H···A
N1–H1**···**F1	0.86	2.35	2.674(2)	130
N1–H1**···**O1	0.86	2.10	2.781(2)	135
C1–H1A**···**O2^(i)^	0.93	2.43	3.355(3)	172
C15–H15**···**O3	0.93	2.54	2.909(3)	104

#### PYRA-2

The 4-(2-(2,4-difluorophenyl) hydrazinyl)-5-methyl-2-tosyl-1H-pyrazol-3(2H)-one (C_17_H_14_F_2_N_4_O_3_ S) crystallizes in the monoclinic space group (*P*2_1_/*n*) (Figure 1b). The dihedral angle of 5.19 (11)° was measured between the pyrazole and fluoro phenyl ring. Intramolecular hydrogen bonds N1–H1···F1, N1–H1···O1, and C15–H15···O3 are observed (Table 2). The crystal structure is stabilized via C1···H1···O2 intermolecular hydrogen bonds (Table 2 and Figure 2). Short intermolecular interactions of the type Cg1···Cg2 (3.519 (1) Å), C5···F1···Cg2 (X···Cg = 3.671 Å; 1-*x*, 2-*y*, 2-*z*; *Y*···*X*···Cg = 74.76 (11)°, and Y···Cg = 3.563 (2). Cg1: N3–N4–C7–C8–C9; Cg2: C1–C6, bonds, and the molecules stabilized via C–H···π interactions and π–π stacking.

### Hirshfeld Surface Analysis

**Figure 3:**
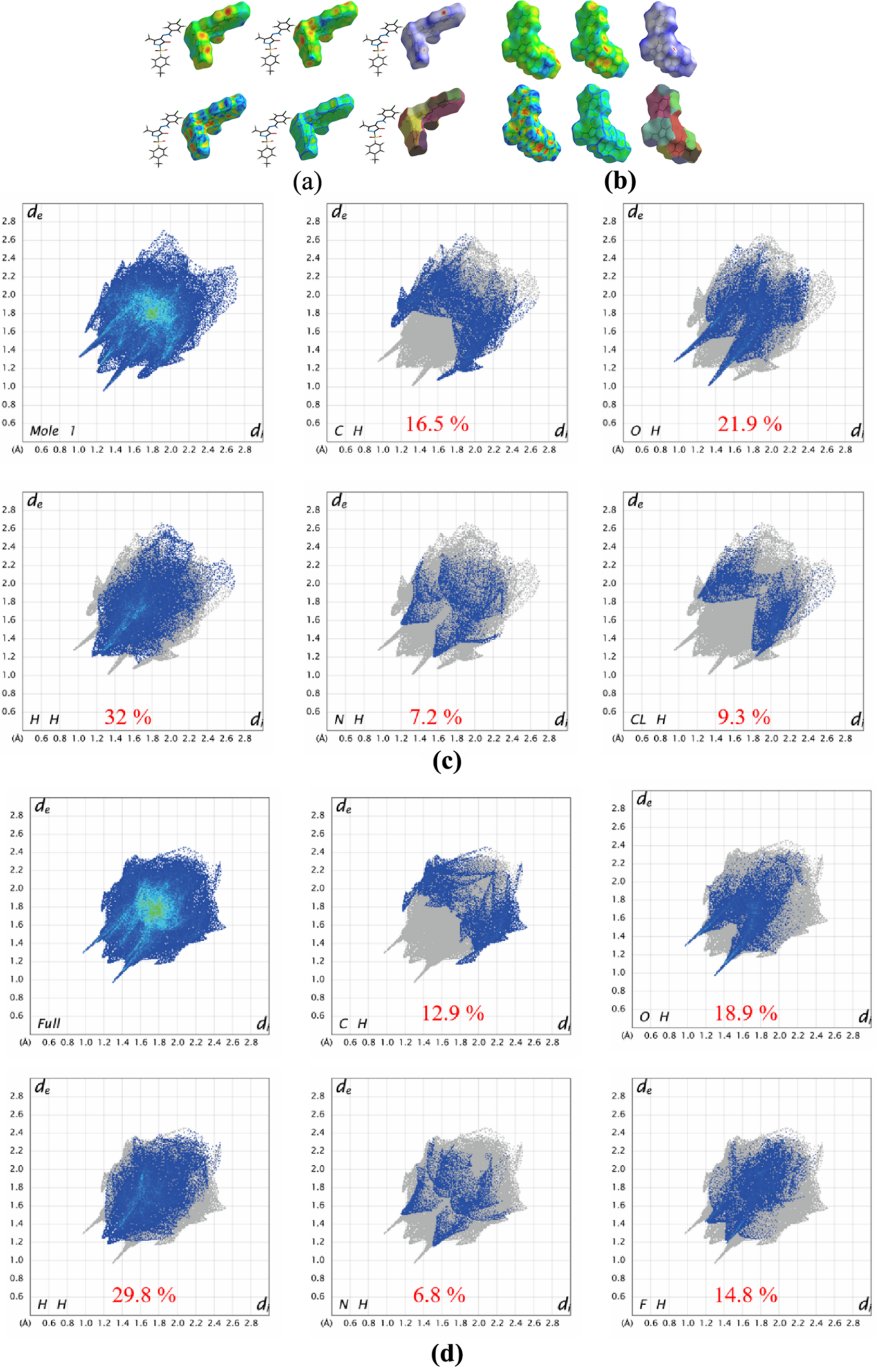
Hirshfeld surface analysis of PYRA-1 (a,c) and PYRA-2 (b,d).

**Figure 4:**
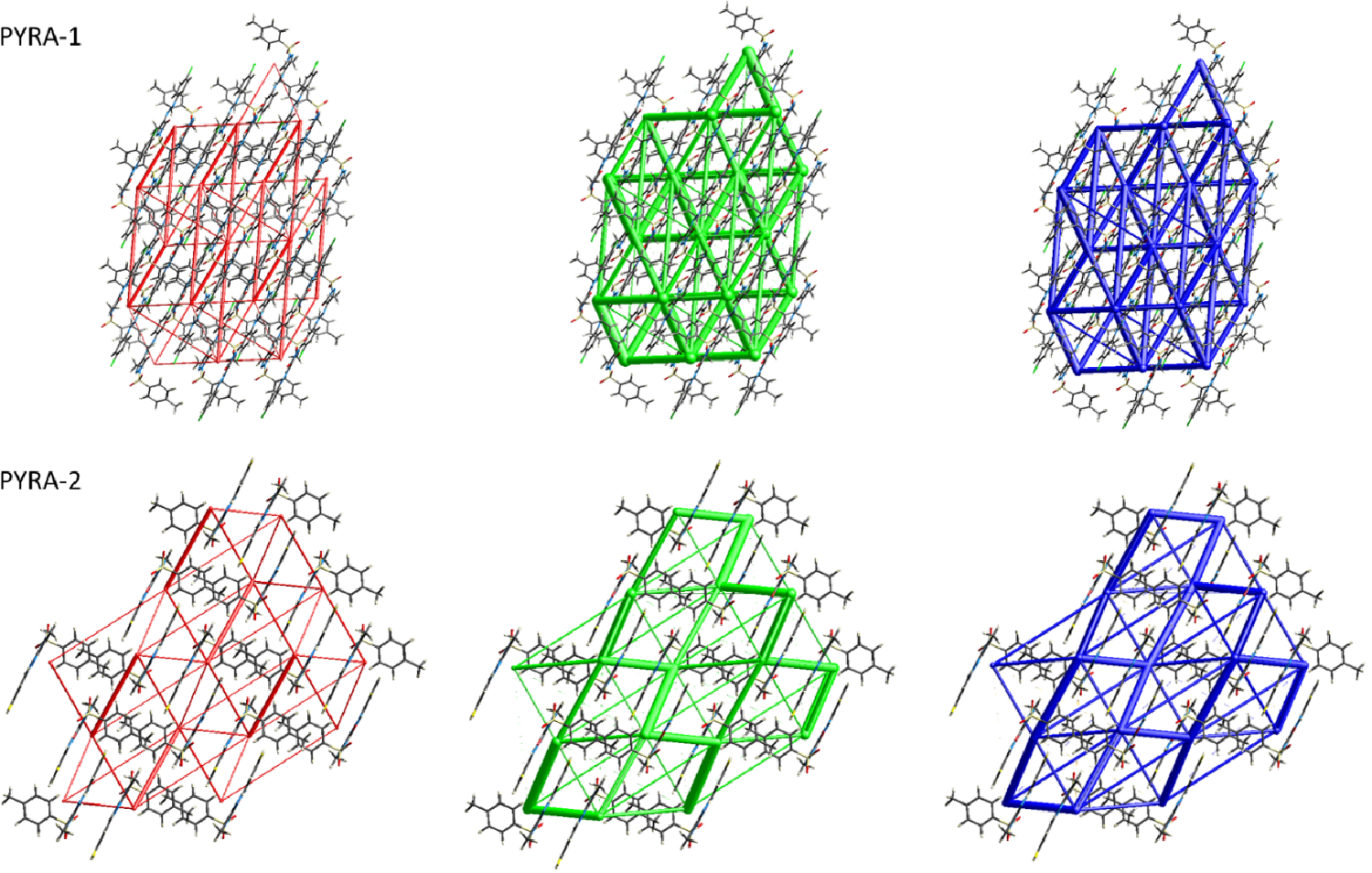
Energy framework analysis of both PYRA-1 and PYRA-2 along the *b*-axis.

In molecular crystals, intermolecular interactions play a crucial role in the creation of supramolecular structures as well as in the packing of crystals. Hirshfeld surface analysis is currently possible to carry out by feeding CIF files generated by crystal structure analysis into applications that are readily available, such as *Crystalexplorer 21.5*, whose main functions may include the display and quantification of various forms of noncovalent interactions between molecules in the crystal phase. Figure 3 depicts different surfaces that were mapped to represent the Hirshfeld surfaces of pyrazole derivatives. In which, both pyrazole derivatives have been mapped over a *d*_norm_, *d*_i_ and *d*_e_, in a transparent manner, allowing visualization of the molecular structure around which these properties were derived. In d_norm_, the surface has three colors: red, blue, and white. The dark red dots represent strong hydrogen bonding locations; the white ones represent connections near the van der Waals separation, and the remaining blue areas represent longer interactions. As noticed, a dark red surface around the S=O group in both compounds creates a hydrogen bonding in the crystal phase. The measurement of intermolecular interactions that were further broken down to study the per-atom interactions present in the structure can be done by using 2D fingerprint plots. For both the compounds, the contributions resulting from various interactions have been examined. The contribution of C···H, O···H, and H···H contacts of PYRA-1 is higher than PYRA-2, whereas the contribution of N···H contacts in PYRA-1 is lower than PYRA-2. Moreover, the halogen substitution in the pyrazole derivatives improves the halogen-based interactions in the crystal phase; for example, the one CL atom in PYRA-1 forms CL···H contacts and their contribution is 9.3%; similarly, the two F atoms in PYRA-2 make F···H contacts and their contribution is 14.8%. The contribution of F···H contacts in PYRA-2 was found to be higher than the C···H, and N···H contacts. Therefore, the halogen substitution in the pyrazole derivatives acts as a significant cohesive force in the crystal packing. The pattern of arrangement of scattered points across fingerprint plots, which revealed a significant variation in the interactions between molecules in pyrazole derivatives, highlights the usefulness of Hirshfeld surfaces and, especially, the inspection of fingerprint plots to facilitate visual screening and quick identification of typical structure-related characteristics via a comprehensive structure examination of intermolecular interactions. Additionally, a 3D graphical representation of the amount of intermolecular interaction energies between pairs of molecules has been examined using energy framework computational analysis. An energy framework is used to visualize the strength of the interaction and the 3D architecture of the crystal packing (Figure 4). Using various crystallographic symmetry operations, the projected interaction energies are shown as colored rods connected by the centroids of molecule pairs over the crystal lattice. Based on the radii of rods, the interaction potency is established. Electrostatic energy (*E*_ele_), exchange-repulsion energy (*E*_dis_), and total interaction energy (*E*_tot_) are, respectively, represented by red, green, or blue rods in an energy framework. The highest total energies of 53.8 kJ/mol (PYRA-1) and −42.6 kJ/mol (PYRA-2) are observed corresponding to the molecular pair along the -x, -y, -z symmetries at 7.12 and 10.03 Å distance. Both the molecules are packed in the unit cell by C–H···O types of interactions. The electrostatic and dispersion energies of −19.1/–83.6 kJ/mol (PYRA-1) and −20.8/–33.1 kJ/mol (PYRA-2) are noticed, which are the second most important contributions to the total energy (Table S1,S2). It is revealed that the dimers stacked along the *b*-axis are relevant energy frameworks, as indicated by the thick cylinders connecting molecules.

### Quantum Chemical Calculations

**Figure 5:**
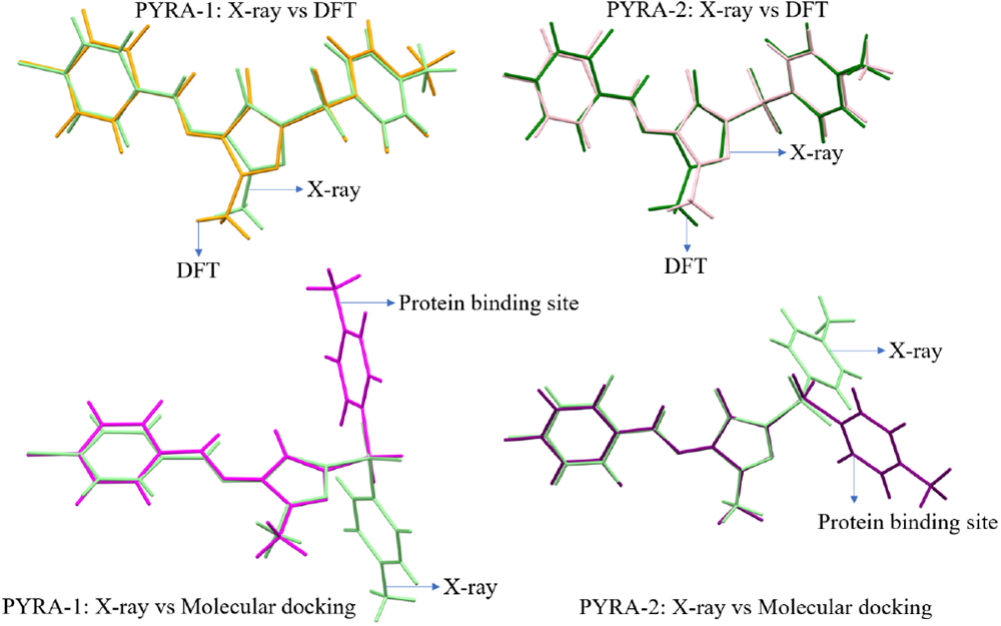
Overlaid view of both pyrazole derivatives of crystal structures and DFT is shown at the top and the crystal structure and protein environment are shown at the bottom.

The optimized and X-ray structures were layered with hydrogen atoms to completely compare them. The outcome of the experiment is based on molecules from the solid state, whereas the computational theory is dependent on individual molecules in the gas phase, which accounts for the visible conformational variation. There are conformational differences between the computed and X-ray structures because the presence of the field that surrounds the crystal and the intermolecular interactions have joined the molecules within the solid state. Good agreement is shown in Figure 5 when the crystallographic and optimized structures of the two compounds are superimposed. The effects of crystal packing are responsible for minor variations in the experimental X-ray structure. Notably, the pyrazole ring shows a quite large deviation than other rings in both compounds due to intramolecular interactions. Similarly, a large conformational deviation is observed between the crystal structure and protein environment at the methyl group and phenyl rings. This is mainly attributed to the binding site effect and strong intermolecular interactions in the protein environment. The structural optimizations of both the molecules were performed in two different solvents (water and DMSO). The results show that both solvent phase optimization are similar. The superimposed solvent-phase and gas-phase structures show RMSDs of 0.196 Å (I) and 0.364 Å (II), respectively (Figure S6). It confirms that conformations of both structures are stable.

**Figure 6:**
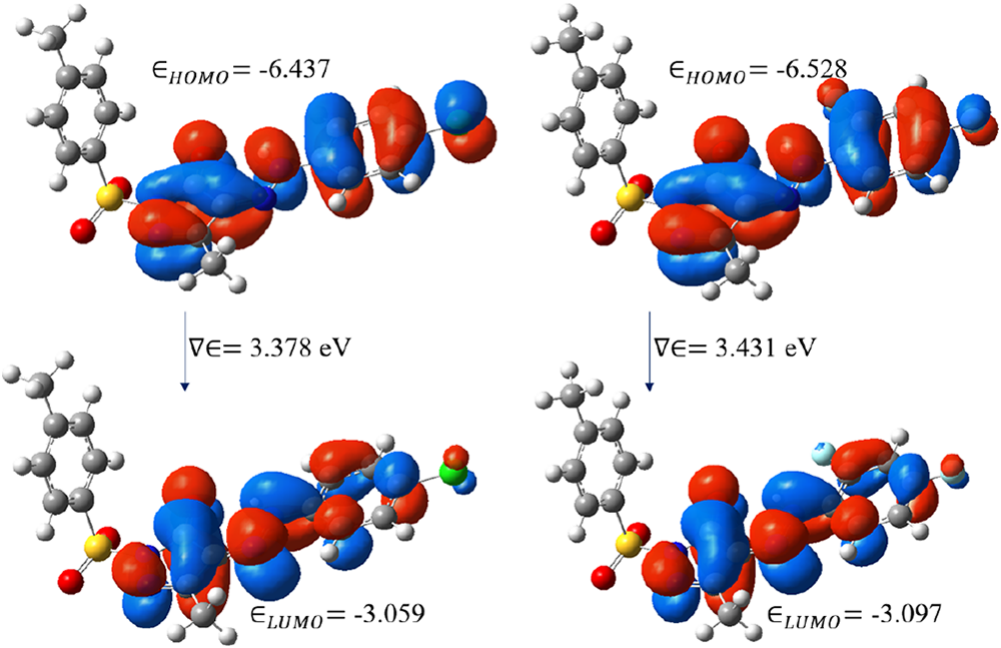
Molecular orbital analyses of PYRA-1 (left) and PYRA-2 (right).

The HOMO energy levels, LUMO, and energy gap were found using DFT to evaluate the electronic properties of molecules, as shown in Table 3. Assessing chemical reactivity and identifying the active sites of molecules during reactions are made easier with the help of quantum chemistry computing. In general, the electron density in LUMO levels is moved toward the electron acceptor, whereas the electron density in HOMO orbitals is largely concentrated on the electron donor. The features of the molecules involved in biological activity can be observed through fluctuations in the energy gap of the molecules. The changes in the energy gap show whether the chemical structure of the molecules influences their electrical characteristics and biological activity. The ionization potential correlates with the HOMO energy, whereas the electron affinity and electronegativity are both linked to the LUMO energy. A molecular stability is greatly influenced by its chemical hardness. From Table 3, the calculated global reactivity properties of PYRA-2 seem to be higher than PYRA-1; however, the values of both pyrazole derivatives are not much varied, it indicates that both compounds are chemically active. The band gap energies of both molecules are less, resulting in charge transfer in the molecule. Furthermore, the chemical properties of both pyrazole compounds carry acceptable values that confirm their biological activity. Figure 6 shows the molecular surfaces, in which the electron densities of HOMO and LUMO are located at pyrazole, fluorobenzene, and dichlorobenzene groups.

**Table 3:** Calculated Global Reactivity Properties of the Molecule

	DFT energy (eV)	
global reactivity descriptors	PYRA-1	PYRA-2
band gap	3.378	3.431
HOMO energy	–6.438	–6.529
LUMO energy	–3.06	–3.098
ionization potential *I* = −*E*_HOMO_	6.438	6.529
electron affinity *A* = −*E*_LUMO_	3.06	3.098
global hardness η = (*I* – *A*)/2	1.689	1.715
electronegativity χ = (*I* + *A*)/2	4.749	4.813
electrophilicity ω = μ^2^/2η, μ = −χ	6.676	6.753

**Figure 7:**
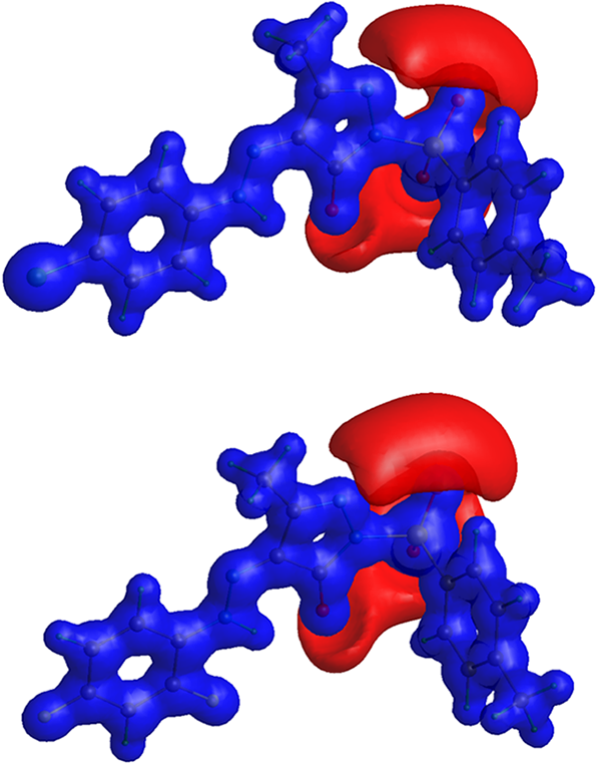
Molecular electrostatic potential map of PYRA-1 and PYRA-2

Understanding chemical reactivity, molecular electrostatic potential (MEP), and electrostatic interactions strongly relies on knowing the charge distribution of the molecule. Atomic charges must be present for the natural population analysis (NPA), which examines the molecule’s electrostatic characteristics. From the spherical charge approximation, the NPA charges are computed. The MPA charge of the sulfur atom possesses a higher positive charge (∼2.22 e) in both the compounds that connects with negatively charged atoms (N, O, and C atoms). The MPA charge of the methyl group carbon atom carries a higher negative charge (−0.60e), whereas the keto group carbon atom in the pyrazole group carries a higher positive charge (∼0.61e). The MPA charge of the chlorine atom in PYRA-1 is very less positive, whereas the MPA charge of florine atoms holds high negative charges in PYRA-2. Furthermore, the MPA charge of the hydrogen atom in amine (N–H) group owns a higher positive charge (0.42e) than other hydrogen atoms in the molecule (Figures S2,S3). The concept of MEP can be used to analyze the distribution of electron density as well as to determine how reactive organic molecules would be in either electrophilic or nucleophilic biological applications. It is made up of many hues that can be seen on maps and are illustrated by various charges. The nucleophilic area is represented by the color blue because it has a positive potential, and thus, has a tendency to evade protons. The portion of the map that is red symbolizes the electrophilic zone and has a negative potential that attempts to bring a proton toward it. The vicinity of the negative potential around sulfonyl and pyrazole groups is due to electronegative atoms in the molecule (Figure 7).

### Molecular Docking Analysis

**Figure 8:**
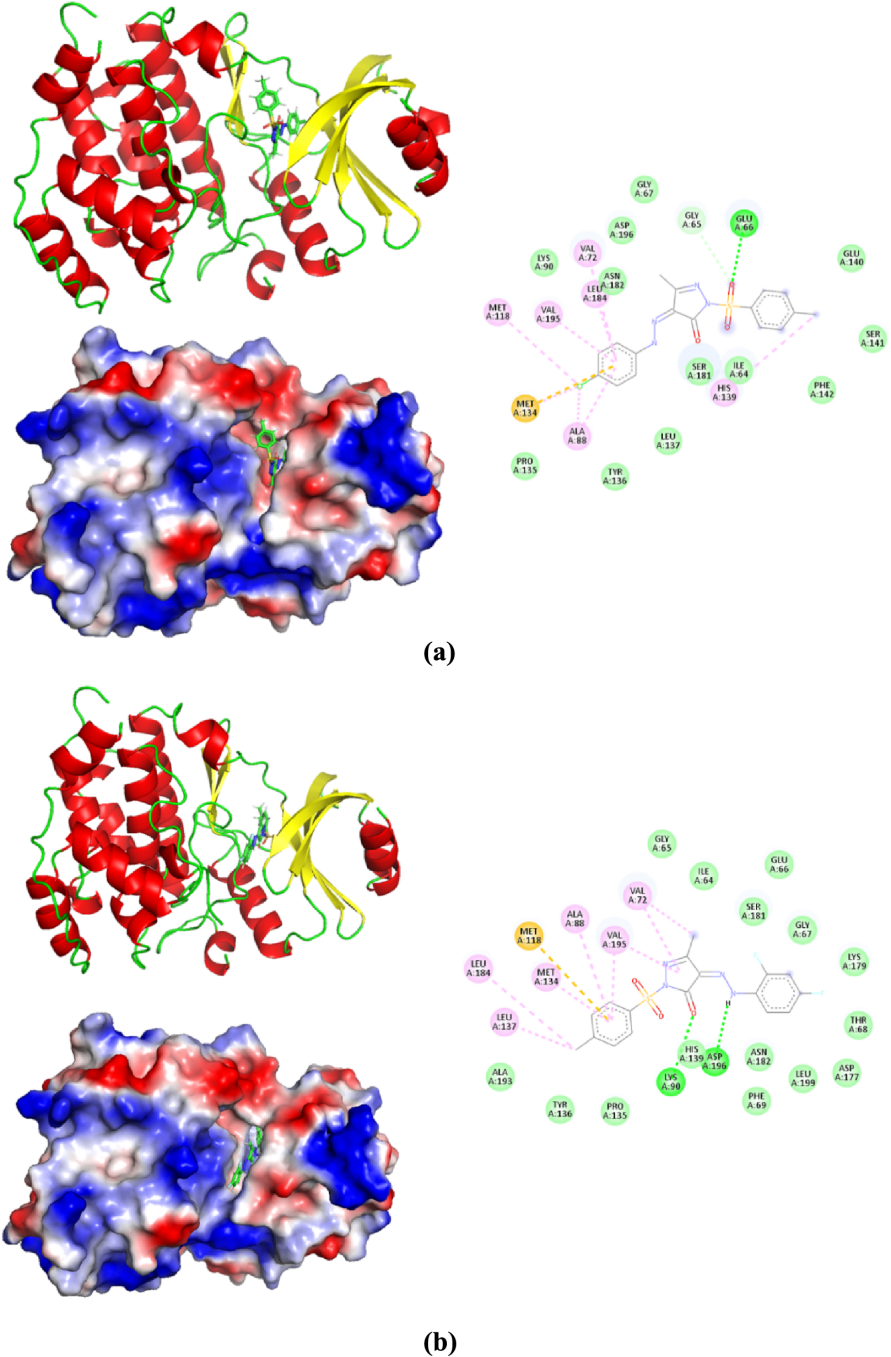
Molecular docking analysis of (a) PYRA-1 and (b) PYRA-2.

Determining the type of binding as well as the binding interactions of the actively docked conformations can help in the development of more effective drug alternatives. The molecular docking research was carried out by utilizing the IFD approach in order to comprehend the binding affinity and intermolecular interactions of PYRA-1 and PYRA-2 with CDC7 kinase. Intermolecular interactions were used to assess the binding potential of both pyrazole compounds. The values of the docking scores for PYRA-1 and PYRA-2 with the CDC7 kinase complexes are −5.421 and −5.884 kcal/mol, respectively. Figure 8 depicts the complexes of intermolecular interactions from a 2D perspective. With the CDC7 kinase binding site residues, the PYRA-1 and PYRA-2 molecules generate hydrophobic, hydrogen bonding, and electrostatic interactions. Particularly, the S=O group of PYRA-1 formed hydrogen bonding with the hydrogen atom of Glu66 at the interaction distance of 2.34 Å. Similarly, the same S=O group forms a carbon hydrogen bonding at the distance of 2.51 Å. The keto group in the pyrazole derivative of PYRA-2 interacts as a conventional hydrogen bond with Lys90 at the distance of 2.71 Å. A similar kind of interaction has also been observed between the amine group of PYRA-2 and Asp196 at 2.47 Å. The π-sulfur-based interaction has noticed between both the compounds and Met134/Met118. Furthermore, PYRA-1 and PYRA-2 appeared to have hydrophobic interactions with Ala88, Val72, Leu184, and Val195. According to the reported crystal structure of CDC7 kinase with inhibitor,^52^ the active site residues are Ala88, Ile64, Met118, Met134, Tyr136, Leu184, and Val195. The superimposed view of the reported inhibitor and our proposed inhibitors shows that all three ligand molecules are present in the same region (active site) of the protein (Figure S7). Therefore, we can confirm that our proposed ligand molecules have potential activity against the CDC7 kinase enzyme. The Connolly surface view of both the pyrazole compounds with CDC7 kinase confirms that the ligand molecule completely occupied the binding cavity of CDC7 kinase and indicates these compounds may act as potential kinase inhibitors.

### Molecular Dynamics and Binding Free Energy Calculation

**Figure 9:**
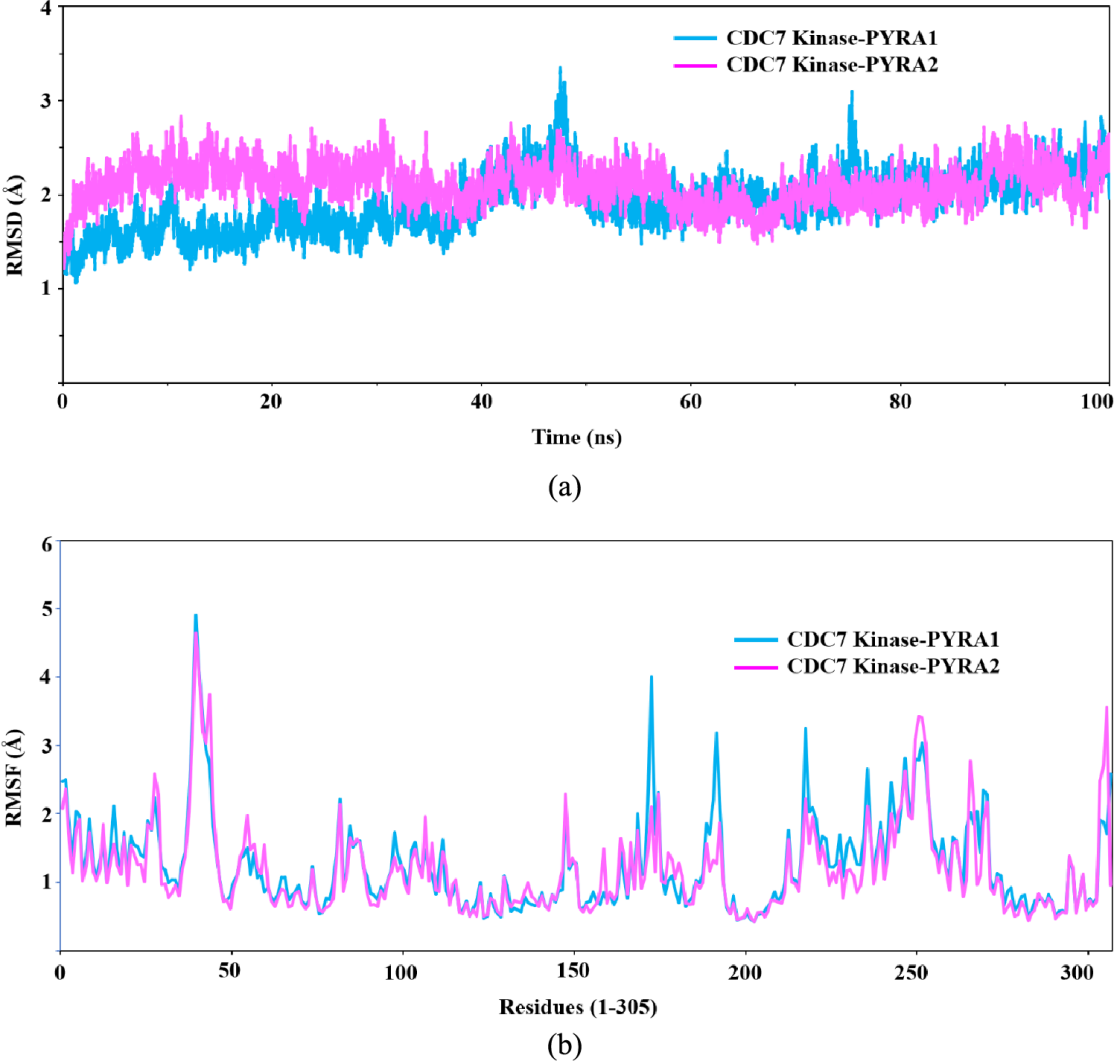
RMSD (a) and RMSF (b) of CDC7 kinase with PYRA-1 and PYRA-2 obtained from MD simulations

**Figure 10:**
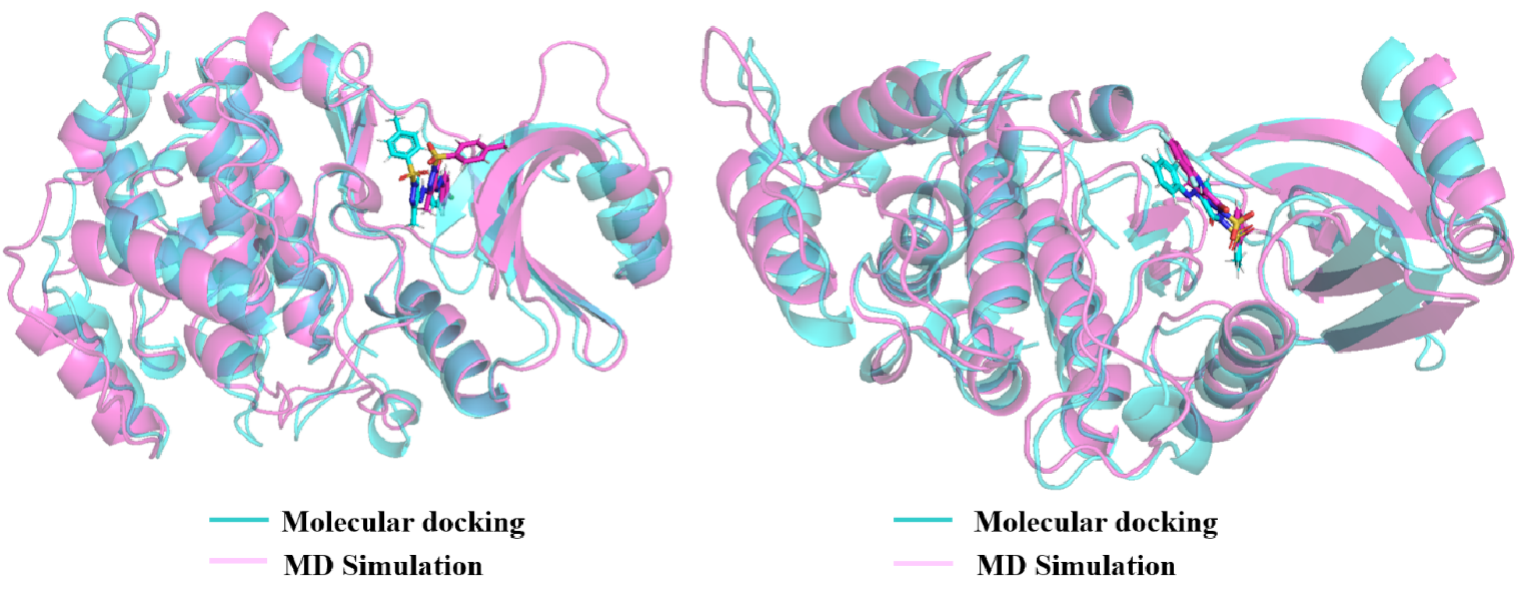
Superimposed view of CDC7 kinase with PYRA-1 and PYRA-2 obtained from docking and MD simulations.

The MD simulation was performed to analyze the stability and binding energy of pyrazole compounds and the residues of the CDC7 kinase enzyme. The best confirmation of both complexes obtained from the docking analysis was taken for the MD simulation. Figure 9a shows the RMSD and root-mean-square fluctuation (RMSF) of both complexes retrieved from the 100-ns MD simulation, which are essential to understand the conformational flexibility, ligand modification in the binding site of CDC7 kinase, and the stability of pyrazole compounds in the CDC7 kinase enzyme. The structural variations that occurred during the ligand–protein interaction is detailed in the RMSD graph. The RMSD plot of protein–ligand complexes have been generated for comparison to assess the stability of their complexes for the interpretation of MD simulation results. From the MD simulation, the RMSDs of PYRA-1– and PYRA-2–CDC7 kinase complexes were analyzed, which gives the deviation of backbone atoms of the enzyme with respect to initial coordinates during the MD simulation. Particularly, the RMSD of the PYRA-2–CDC7 kinase complex quickly achieves the stable equilibrium within few nanoseconds and maintains their stability at the end of 100 ns while the PYRA-1–CDC7 kinase complex has not achieved a stable conformation until the end of 80 ns. The RMSD plot gives two zigzag conformations at around 50 and 80 ns, respectively. However, the average RMSD of PYRA-1–CDC7 kinase complex is found to be lower than the PYRA-2–CDC7 kinase complex, whose values are 1.91 and 2.10 Å, respectively. This small deviation in the RMSD plot suggested the spatial arrangement of the active site to accommodate the ligand molecule. Furthermore, to understand the flexibility and the mobility of each amino acid with respect to the ligand molecule in the binding site of CDC7 kinase, RMSF is an important parameter (Figure 9b). In both complexes, the RMSF of *C*-terminal residues possess higher fluctuation than the *N*-terminal residues. Also, the loops and turns of the CDC7 kinase give larger flexibility on comparing with α-helix and β-sheets in the enzyme. Importantly, the active sites residues at Lys90, Val72, Ala88, Asp196, Met134, Leu184, and Val195 exhibit smaller RMSF values, which indicates that the binding site residues are not much fluctuated due to the intermolecular interactions. Furthermore, both the complexes were subjected to superimpose with the corresponding docking complexes to understand the ligand flexibility with respect to the dynamical behavior of the protein environment. From the overlaid (Figure 10), the phenyl group of PYRA-1 is completely deviated, and the fluorobenzene and pyrazole groups were found to retain their conformation in the active site at the end of 100 ns. However, the conformation of PYRA-2 is exactly engaged in the active site throughout the MD simulation. The stability of the interaction between the protein–ligand complex strongly depends on the uniformity of the spatial orientation of the ligand and the ideal distance from the binding cavity of the protein in order to preserve the dynamic stability of the ligand at the binding pocket of the protein. Thus, the intermolecular interaction was also examined, in the PYRA-1–CDC7 kinase, and the conformational modification of ligand molecule at the end of simulation leads to rearrangement of the intermolecular interaction. However, the chlorine atom forms an alkyl-based hydrophobic interaction and it maintains in the MD with a distance of 3.5 Å. Ile64 creates a new hydrogen bonding with amine group of PYRA-1 with the distance 2.66 Å. In the PYRA-2–CDC7 kinase, the keto group in the pyrazole group is highly engaged in the hydrogen bonding with Lys90 and Asp196 in both docking and dynamics, with the distance of 2.7 and 2.1 Å, respectively. It clearly confirms that PYRA-2 is a more potential inhibitor than PYRA-1 against CDC7 kinase. Table S3 and Figure S7 show the complete intermolecular interactions between CDC7 kinase and PYRA-1/PYRA-2. This rearrangement, missing of interaction, and conformational modification of ligand molecule are reflected in the binding energy calculation. The calculated binding free energy of PYRA-1–CDC7 kinase complex is −36.71 kcal/mol in the MD, while the value obtained from docking is −39.22 kcal/mol. Similarly, the binding energy of PYRA-2–CDC7 kinase is −45.18 kcal/mol for MD, whereas −59.11 kcal/mol for docking. On comparison of both the complexes, there is no much binding energy difference in the PYRA-2 complex due to strong intermolecular interactions and stability of PYRA-2 in the active site of the CDC7 kinase enzyme.

### Non-Covalent Interaction Analysis

**Figure 11:**
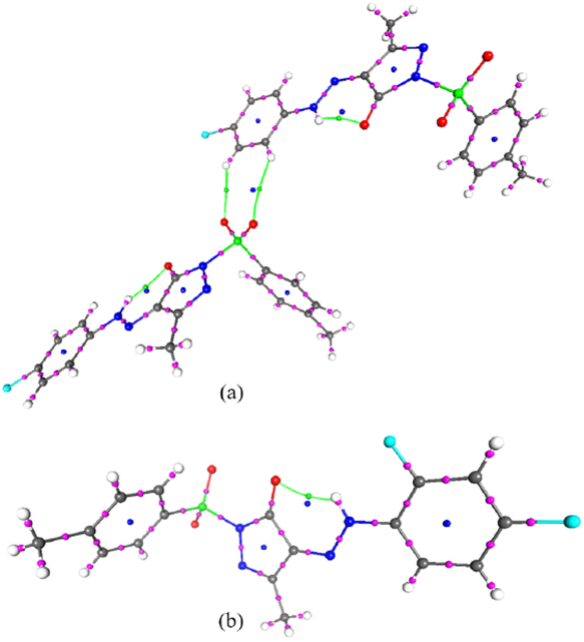
BCP map of (a) PYRA-1 and (b) PYRA-2.

**Figure 12:**
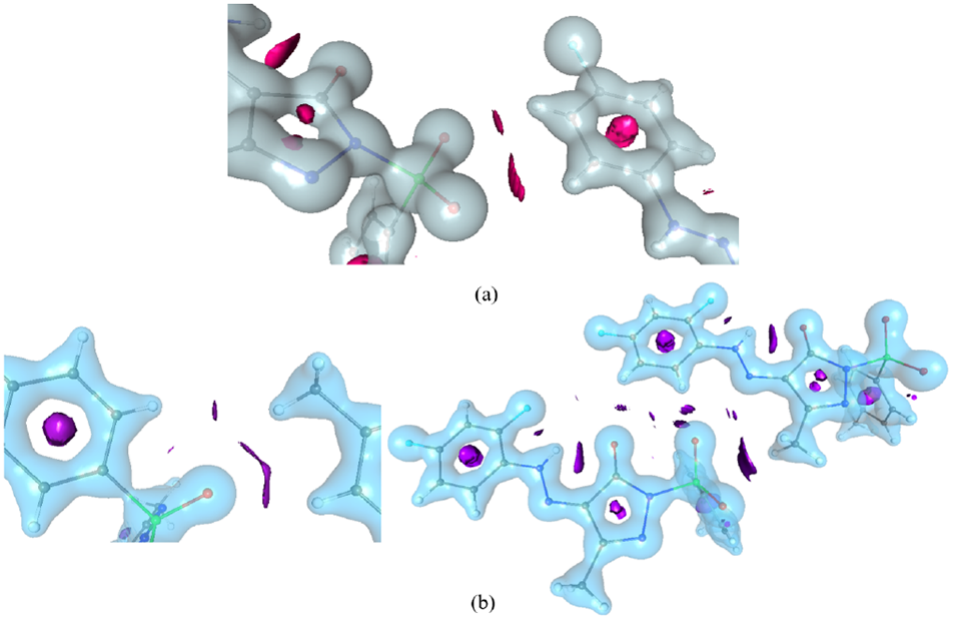
NCI isosurface maps of (a) PYRA-1 and (b) PYRA-2

Recent studies have shown noncovalent interactions, more specifically, those involving H-bonds, the steric impact, and van der Waals interactions. It is a computational-based theoretical method to describe the molecular system by calculating electron density and their derivatives.^47,48^ In general, noncovalent interactions are thought of as weak interactions and are highly helpful in understanding how molecules behave in relation to their chemical and biological activities. Similarly, QTAIM analysis also utilizes electron density and has emerged as a popular method to look for many types of molecular interactions. QTAIM offers an exclusive opportunity to comprehend a particular area of the system based on the physical characteristics of that system based on zero-flux in the electron density gradient field. The nature of chemical bonds and their interaction categories and interaction strengths can all be classified using the electron density obtained from the QTAIM analysis. Based on results, both pyrazole derivatives are forming weak C–H···O type of intermolar interactions and N–H···O type of intramolecular interactions in the crystal phase. A (3, −1) type of critical point search was conducted to observe all kinds of noncovalent interactions in the crystal phase as well as in the protein environment (Figures 11 and S4). Particularly, the topological properties of sulfone group oxygen with C4B–H4B and C5B–H5B carries weak electron density and Laplacian of electron density, the values are 0.08/0.026 eÅ^–3^ and 0.944/0.317 eÅ^–5^, respectively. Similar kinds of interactions were noticed in molecule 2, the average electron density of C–H···O interaction is 0.059 eÅ^–3^ and their Laplacian of electron density is 0.735 eÅ^–5^. In the CDC7 kinase enzyme environment, PYRA-1 and PYRA-2 form strong intermolecular interactions, with a detailed discussion in the Molecular Docking section. Here, the topological properties of those noncovalent interactions were calculated. In which, the chlorine atom of PYRA-1 possesses three bond paths and critical points with Ala88 and Met118, the average values are 0.114 eÅ^–3^ and 1.07 eÅ^–5^, respectively. Moreover, the sulfone group oxygen forms a bond path to Gly65 and Glu66, values are 0.069/0.933 eÅ^–3^ and 0.843/1.11 eÅ^–5^, respectively. A similar trend was noticed in PYRA-2, where the band path and critical point were seen between the sulfone group oxygen and Lys92/Met118, whose values were 0.063/0.059 eÅ^–3^ and 0.742/0.733 eÅ^–5^, respectively. The topological properties of all the weak interactions in both crystal phase and protein environment were found to be less and positive Laplacian values indicate that the interactions are ***closed-shell interactions***. The NCI isosurface map was generated for noncovalent interactions obtained in both crystal and protein environments (Figures 12 and S5) which shows the NCI isosurface between two interacting atoms that confirms the strength of the interaction. Furthermore, studies showed the electrostatic interaction energy also plays a vital role in the analysis of stability and strength of intermolecular interactions in the protein environment.^39,46,58^ The calculated total electrostatic interaction energy of the CDC7 kinase–PYRA-2 complex (−58.08 kcal/mol) carries higher energy than that of the CDC7 kinase–PYRA-1 complex (−40.03 kcal/mol). This is exactly correlating with the binding energy obtained from MD analysis. However, this total electrostatic energy is found to be lower than the recently reported JNK3 and ACE2 complexes as well as higher than the SARS Cov-19 Mpro, HER2 complexes.^39,58^ Therefore, the electrostatic interaction energy calculation is also an additional support to evaluate the strength of noncovalent interactions.

## Summary and Conclusion

In this work, two pyrazole derivatives, PYRA-1 and PYRA-2, were characterized with 3D molecular structures (X-ray diffraction and DFT) and screened (*in silico* molecular docking and MD) against the CDC7 kinase target. In addition, their crystal structure arrangements were analyzed using Hirshfeld surfaces analysis, noncovalent interactions, and quantum theory of atoms in molecules. The 3D arrangement of both the molecules in the crystal structures were stabilized through C···H···O intermolecular hydrogen bonds and the weak intermolecular interactions (C–H···π and π–π stacking). The in silico screening confirms that PYRA-2 shows more potent toward the CDC7 kinase target than the PYRA-1 molecule.

## Data Availability

The crystallographic data for the structure reported in this paper have been deposited with the Cambridge Crystallographic Data Centre as supplementary publication no. CCDC for PYRA-1 and PYRA-2 are 2265839 and 2265838, respectively. Copies of the data can be obtained free of charge by application to CCDC,12 Union Road, Cambridge, CB21EZ, U.K. (fax:(+44)1223–336–033, e-mail:deposit@ccdc.cam.ac.uk)

## Supporting Information Available

Scheme of the reaction in Scheme 1. The spectral details FTIR (**Figure** S1) of EAS3 and EAS8; Interaction energies of PYRA-1 (Table S1) and PYRA-2 (Table S2); Intermolecular interactions observed after 100 ns of MD simulations (Table S3); Atomic charges of PYRA-1 (**Figure** S2) and PYRA-2 (**Figure** S3); BCP map of PYRA-1 and PYRA-2 (**Figure** S4); NCI isosurface of protein–ligand complexes (**Figure** S5); The superimposed view of gas phase and solution phase (**Figure** S6); 2D view of intermolecular interactions observed from 100 ns of MD simulations (**Figure** S7) (PDF)
The crystallographic data for the structure reported in this paper have been deposited with the Cambridge Crystallographic Data Centre as supplementary publication no. CCDC for PYRA-1 and PYRA-2 are 2265839 and 2265838, respectively. Copies of the data can be obtained free of charge by application to CCDC,12 Union Road, Cambridge, CB21EZ, U.K. (fax: (+44)1223-336-033, e-mail: deposit@ccdc.cam.ac.uk)
