# Supplementary material for: A Structural and In Silico Investigation of Potential CDC7 Kinase Enzyme Inhibitors
Source: ACS Omega. 2023 Nov 27;8(49):47187–200. doi: 10.1021/acsomega.3c07059 (PMC10719926; doi:10.1021/acsomega.3c07059)
Supplement: Supplementary file 1 — ao3c07059_si_003.pdf [file ao3c07059_si_003.pdf]

A structural and *in silico* investigation of potential CDC7 kinase enzyme inhibitors

Mohanbabu Mookkan[1$], Saravanan Kandasamy[2$], Abdel-Basit Al-Odayni[3], Naaser Ahmed Yaseen Abduh[4], Sugarthi Srinivasan[5], Bistuvalli Chandrashekara Revannasidappa[6], Vasantha Kumar[7], Kalaiarasi Chinnasamy[8], Sanmargam Aravindhan[1] and Madan Kumar Shankar[9*]

[1]Department of Physics, Presidency College (Autonomous), University of Madras, Chennai 600 005, India
[2]Faculty of Chemistry, University of Warsaw, Ludwika Pasteura 1, Warsaw, 02-093, Poland
[3]Department of Restorative Dental Science, College of Dentistry, King Saud University, P. O. Box 60169, Riyadh 1145, Saudi Arabia.
[4]Department of Chemistry, College of Science, King Saud University, P.O. Box 2455, Riyadh 11451, Saudi Arabia.
[5]Department of Physics and Nanotechnology, SRM Institute of Science and Technology, Kattankulathur, Kancheepuram, India
[6]Department of Pharmaceutical Chemistry, NGSM Institute of Pharmaceutical Sciences of Nitte - Deemed to be University, Paneer, Deralakatte, Bangalore 575018, Karnataka, India.
[7]Department of P.G. Chemistry, Sri Dharmasthala Manjunatheshwara College (Autonomous), Ujire-574240, India
[8]Molecular Biophysics Unit, Indian Institute of Science, Bangalore 560 012, India
[9]Department of Chemistry-BMC, University of Uppsala, Husargatan 3, Uppsala, 75237, Sweden

[$] equal contribution
* corresponding author: madan.shankar@kemi.uu.se

Spectral details of EAS3 and EAS8

The melting point of the compound EAS3 and EAS8 is 114 °C and 116 °C, respectively. The FTIR of EAS3 and EAS8 are shown below (**Figure** S1 (a) and (b)).

PYRA-1: FTIR (KBr, γ_{max}, cm^{-1}): 3339 (N-H), 3080 (=C-H), 2943 (-C-H), 2883 (-C-H), 1680 (C=O), 1589 (C=N), 1563 (C=C), 1395 and 1218 (S=O), 819 (C-F); ^{1}H NMR (400 MHz, DMSO-d_6, δ in ppm): 12.88 (1H, s, NH), 7.92 (2H, d, J = 8.4 Hz, Ar-H ortho to SO_2), 7.46 (2H, d, J = 8.4 Hz, Ar-H ortho to CH_3), 7.28 (2H, d, J = 8 Hz, Ar-H of 4-chlorophenyl), 7.05 (2H, d, J = 8 Hz, Ar-H of 4-chlorophenyl), 2.41 (3H, s, Ar-CH_3), 2.16 (3H, s, pyrazolyl-CH_3).

PYRA-2: FTIR (KBr, γ_{max}, cm^{-1}): 3335 (N-H), 3071 (=C-H), 2940 (-C-H), 2880 (-C-H), 1682 (C=O), 1585 (C=N), 1560 (C=C), 1390 and 1215 (S=O), 1156 (C-F); ^{1}H NMR (400 MHz, DMSO-d_6, δ in ppm): 12.90 (1H, s, NH), 7.90 (2H, d, J = 8.4 Hz, Ar-H ortho to SO_2), 7.50-7.40 (4H, m, Ar-H ortho to CH_3 and Ar-H of 2,4-difluorophenyl), 7.15-7.10 (1H, m, Ar-H of 2,4-difluorophenyl), 2.40 (3H, s, Ar-CH_3), 2.15 (3H, s, pyrazolyl-CH_3).

IR spectrum of **PYRA-1** shows N-H stretching band at 3339 cm^{-1} and carbonyl stretching band at 1680 cm^{-1} is in confirmation for the product formation. It further shows S=O asymmetric and symmetric stretching bands at 1395 and 1218 cm^{-1} respectively. It also shows C-Cl stretching band at 819 cm^{-1}. In the IR spectrum of IR spectrum of **PYRA-2**, N-H stretching band was observed at 3335 cm^{-1} and carbonyl stretching band at 1682 cm^{-1}. The s S=O asymmetric and symmetric stretching bands observed at 1390 and 1215 cm^{-1} respectively. It also shows C-F stretching band at 1156 cm^{-1}.

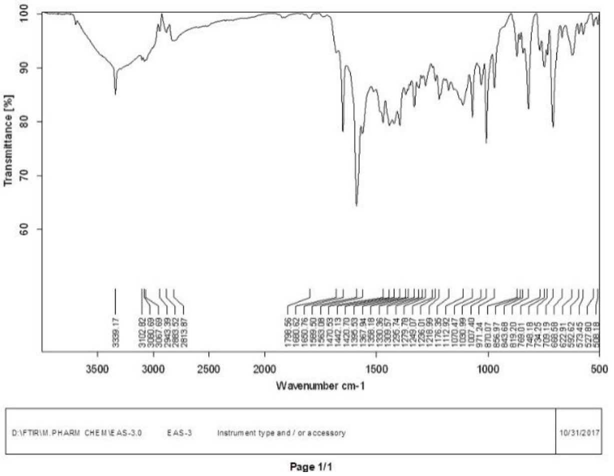

(a)

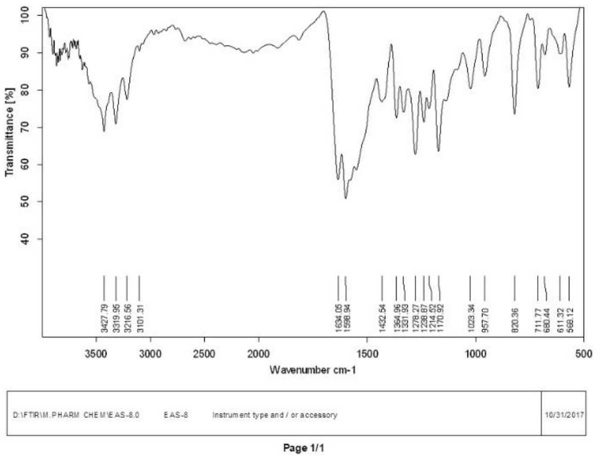

(b)

Figure S1: Spectral graphs: FTIR (a) PYRA-1 and (b) PYRA-2.

Table S1: Interaction Energies (kJ/mol) of PYRA-1

R is the distance between molecular centroids (mean atomic position) in Å.

Total energies, only reported for two benchmarked energy models, are the sum of the four energy components, scaled appropriately (see the scale factor table below)

	N	Symop	R	Electron Density	E_ele	E_pol	E_dis	E_rep	E_tot
	2	x, y, z	8.18	B3LYP/6-31G(d,p)	-6.8	-2.8	-9.5	6.9	-13.3
	1	-x, -y, -z	12.62	B3LYP/6-31G(d,p)	-1.1	-0.1	-0.4	0.0	-1.5
	1	-x, -y, -z	7.12	B3LYP/6-31G(d,p)	-5.8	-3.9	-83.6	45.5	-53.8
	1	-	11.81	B3LYP/6-31G(d,p)	0.0	nan	0.0	0.0	nan
	1	-	16.51	B3LYP/6-31G(d,p)	-0.0	-0.0	-0.0	0.0	-0.0
	1	-	10.30	B3LYP/6-31G(d,p)	-0.1	-0.0	-0.0	0.0	-0.1
	2	x, y, z	18.54	B3LYP/6-31G(d,p)	-0.1	-0.0	-0.2	0.0	-0.3
	1	-x, -y, -z	15.70	B3LYP/6-31G(d,p)	-0.3	-0.1	-0.2	0.0	-0.6
	1	-x, -y, -z	10.23	B3LYP/6-31G(d,p)	-19.1	-6.1	-31.9	20.9	-39.5
	2	x, y, z	16.10	B3LYP/6-31G(d,p)	0.5	-0.0	-0.1	0.0	0.5

	1	-	17.52	B3LYP/6-31G(d,p)	-0.1	-0.0	-0.2	0.0	-0.3
	1	-	12.73	B3LYP/6-31G(d,p)	-2.5	-0.3	-6.9	0.0	-8.9
	1	-	15.86	B3LYP/6-31G(d,p)	-6.8	-2.8	-9.5	6.9	-13.3
	2	x, y, z	16.65	B3LYP/6-31G(d,p)	0.5	-0.0	-0.5	0.0	0.1
	1	-	10.29	B3LYP/6-31G(d,p)	-0.5	-0.1	-0.2	0.0	-0.7
	1	-	10.77	B3LYP/6-31G(d,p)	-1.1	-0.1	-0.4	0.0	-1.5
	1	-	15.12	B3LYP/6-31G(d,p)	-0.8	-0.0	-0.1	0.0	-1.0
	1	-	10.77	B3LYP/6-31G(d,p)	0.0	-0.0	-0.1	0.0	-0.1
	1	-	16.49	B3LYP/6-31G(d,p)	-0.1	-0.0	-0.0	0.0	-0.1
	2	x, y, z	15.60	B3LYP/6-31G(d,p)	1.8	-0.3	-6.9	0.0	-4.4
	2	x, y, z	14.25	B3LYP/6-31G(d,p)	-0.4	-0.1	-0.2	0.0	-0.6
	1	-x, -y, -z	16.98	B3LYP/6-31G(d,p)	0.5	-0.0	-0.1	0.0	0.4
	1	-x, -y, -z	8.71	B3LYP/6-31G(d,p)	-5.2	-0.7	-54.7	30.3	-34.9
	1	-	15.90	B3LYP/6-31G(d,p)	-0.0	-0.0	-0.0	0.0	-0.0
	1	-	12.72	B3LYP/6-31G(d,p)	-6.8	-2.8	-9.5	6.9	-13.3
	1	-	7.30	B3LYP/6-31G(d,p)	0.5	-0.0	-0.1	0.0	0.4
	1	-x, -y, -z	9.83	B3LYP/6-31G(d,p)	-4.6	-1.3	-49.8	24.9	-33.8
	1	-	12.28	B3LYP/6-31G(d,p)	0.3	-0.0	-0.5	0.0	-0.1
	1	-	18.45	B3LYP/6-31G(d,p)	-0.0	-0.0	-0.0	0.0	-0.1
	1	-	16.25	B3LYP/6-31G(d,p)	-0.1	-0.0	-0.1	0.0	-0.2
	1	-	10.82	B3LYP/6-31G(d,p)	0.6	-0.0	-0.1	0.0	0.5
	1	-	7.22	B3LYP/6-31G(d,p)	-5.8	-3.9	-83.6	45.5	-53.8
	1	-x, -y, -z	16.14	B3LYP/6-31G(d,p)	1.7	-0.1	-0.2	0.0	1.5
	1	-x, -y, -z	15.55	B3LYP/6-31G(d,p)	-1.5	-0.1	-0.3	0.0	-1.9
	1	-	9.70	B3LYP/6-31G(d,p)	0.2	-0.0	-0.0	0.0	0.2
	1	-	18.09	B3LYP/6-31G(d,p)	-19.1	-6.1	-31.9	20.9	-39.5
	1	-x, -y, -z	23.28	B3LYP/6-31G(d,p)	-0.1	-0.0	-0.1	0.0	-0.2
	1	-x, -y, -z	15.34	B3LYP/6-31G(d,p)	-0.2	-0.0	-0.2	0.0	-0.4
	1	-	19.16	B3LYP/6-31G(d,p)	-0.0	-0.0	-0.0	0.0	-0.0

	1	-	17.83	B3LYP/6-31G(d,p)	-0.5	-0.0	-0.1	0.0	-0.6
	1	-	11.59	B3LYP/6-31G(d,p)	0.4	-0.0	-0.1	0.0	0.3
	1	-x, -y, -z	14.92	B3LYP/6-31G(d,p)	1.0	-0.0	-0.4	0.0	0.7
	1	-	16.54	B3LYP/6-31G(d,p)	0.1	-0.0	-0.0	0.0	0.0
	1	-	19.70	B3LYP/6-31G(d,p)	0.3	-0.0	-0.0	0.0	0.3
	1	-	19.17	B3LYP/6-31G(d,p)	-0.1	-0.0	-0.1	0.0	-0.2
	1	-	16.20	B3LYP/6-31G(d,p)	0.3	-0.0	-0.0	0.0	0.3
	1	-	11.06	B3LYP/6-31G(d,p)	-5.2	-0.7	-54.7	30.3	-34.9
	1	-	14.35	B3LYP/6-31G(d,p)	0.2	-0.0	-0.0	0.0	0.2
	1	-	19.08	B3LYP/6-31G(d,p)	-4.6	-1.3	-49.8	24.9	-33.8

Scale factors for benchmarked energy models
See Mackenzie et al. IUCrJ (2017)

Energy Model	k_ele	k_pol	k_disp	k_rep
CE-HF ... HF/3-21G electron densities	1.019	0.651	0.901	0.811
CE-B3LYP ... B3LYP/6-31G(d,p) electron densities	1.057	0.740	0.871	0.618

Table S2: Interaction Energies (kJ/mol) of PYRA-2

R is the distance between molecular centroids (mean atomic position) in Å.

Total energies, only reported for two benchmarked energy models, are the sum of the four energy components, scaled appropriately (see the scale factor table below)

	N	Symop	R	Electron Density	E_ele	E_pol	E_dis	E_rep	E_tot
	2	x+1/2, -y+1/2, z+1/2	11.03	B3LYP/6-31G(d,p)	-8.9	-3.3	-9.0	9.3	-13.9
	2	-x+1/2, y+1/2, -z+1/2	6.93	B3LYP/6-31G(d,p)	-7.9	-5.2	-47.1	23.9	-38.5
	2	x+1/2, -y+1/2, z+1/2	8.08	B3LYP/6-31G(d,p)	-1.5	-0.6	-6.8	1.0	-7.3

	2	x, y, z	14.86	B3LYP/6-31G(d,p)	-0.5	-0.1	-0.3	0.0	-0.8
	2	x+1/2, -y+1/2, z+1/2	12.98	B3LYP/6-31G(d,p)	-5.4	-0.5	-9.9	0.0	-14.7
	2	x+1/2, -y+1/2, z+1/2	12.53	B3LYP/6-31G(d,p)	0.6	-0.1	-1.0	0.0	-0.3
	2	-x+1/2, y+1/2, -z+1/2	14.00	B3LYP/6-31G(d,p)	-2.2	-0.3	-0.9	0.0	-3.4
	2	x, y, z	17.17	B3LYP/6-31G(d,p)	0.6	-0.0	-0.4	0.0	0.3
	2	x+1/2, -y+1/2, z+1/2	16.12	B3LYP/6-31G(d,p)	-0.3	-0.0	-0.3	0.0	-0.7
	2	x, y, z	8.60	B3LYP/6-31G(d,p)	-15.7	-5.2	-26.7	22.6	-29.8
	1	-x, -y, -z	15.19	B3LYP/6-31G(d,p)	-2.3	-0.3	-1.0	0.0	-3.6
	2	x+1/2, -y+1/2, z+1/2	14.99	B3LYP/6-31G(d,p)	-0.2	-0.1	-0.4	0.0	-0.6
	1	-x, -y, -z	7.17	B3LYP/6-31G(d,p)	-7.0	-1.7	-21.8	9.5	-21.7
	1	-x, -y, -z	10.03	B3LYP/6-31G(d,p)	-20.8	-8.0	-33.1	22.8	-42.6
	2	-x+1/2, y+1/2, -z+1/2	10.44	B3LYP/6-31G(d,p)	-1.8	-0.8	-22.4	6.8	-17.8
	2	-x+1/2, y+1/2, -z+1/2	15.36	B3LYP/6-31G(d,p)	0.7	-0.1	-0.2	0.0	0.5
	2	x, y, z	15.56	B3LYP/6-31G(d,p)	0.1	-0.0	-0.3	0.0	-0.1
	2	x, y, z	17.78	B3LYP/6-31G(d,p)	-0.2	-0.0	-0.3	0.0	-0.5
	1	-x, -y, -z	8.31	B3LYP/6-31G(d,p)	-4.1	-0.7	-58.8	25.3	-40.5
	1	-x, -y, -z	10.87	B3LYP/6-31G(d,p)	-0.9	-0.1	-2.9	0.2	-3.5
	2	-x+1/2, y+1/2, -z+1/2	18.55	B3LYP/6-31G(d,p)	-0.3	-0.0	-0.1	0.0	-0.4
	2	-x+1/2, y+1/2, -z+1/2	14.11	B3LYP/6-31G(d,p)	0.3	-0.0	-0.2	0.0	0.1
	1	-x, -y, -z	15.32	B3LYP/6-31G(d,p)	0.7	-0.1	-1.6	0.0	-0.7
	2	-x+1/2, y+1/2, -z+1/2	16.03	B3LYP/6-31G(d,p)	0.2	-0.0	-0.3	0.0	-0.1
	1	-x, -y, -z	18.99	B3LYP/6-31G(d,p)	-0.1	-0.0	-0.2	0.0	-0.3
	1	-x, -y, -z	15.18	B3LYP/6-31G(d,p)	0.7	-0.0	-0.5	0.0	0.3

Scale factors for benchmarked energy models
See Mackenzie et al. IUCrJ (2017)

Energy Model	k_ele	k_pol	k_disp	k_rep

CE-HF ... HF/3-21G electron densities	1.019	0.651	0.901	0.811
CE-B3LYP ... B3LYP/6-31G(d,p) electron densities	1.057	0.740	0.871	0.618

Table S3: Intermolecular interactions observed after 100ns of MD simulation

**PYRA-1 in MD simulation**		
Ligand-H1...ILE24:O	2.664	Conventional Hydrogen Bond
Ligand...GLU26:H	2.841	Pi-Donor Hydrogen Bond
Ligand...VAL32:HG21	2.722	Pi-Sigma
Ligand-Cl1...ALA48	3.719	Alkyl
Ligand-Cl1...MET94	3.952	Alkyl
Ligand-C17...LYS23	4.842	Alkyl
**PYRA-2 in MD simulation**		
Ligand:F1...THR28:H	2.481	Conventional Hydrogen Bond;Halogen (Fluorine)
Ligand:O1...LYS50:HZ1	2.746	Conventional Hydrogen Bond
Ligand:O2...LYS50:HZ1	1.943	Conventional Hydrogen Bond
Ligand:O1...LYS50:HZ3	2.666	Conventional Hydrogen Bond
Ligand:H1...ASP156:OD2	2.118	Conventional Hydrogen Bond
Ligand:O1...LYS50:HE3	2.738	Carbon Hydrogen Bond
Ligand:F1...ASP156:OD2	3.064	Halogen (Fluorine)
Ligand...MET78:SD	5.018	Pi-Sulfur
Ligand:C17...VAL155	4.860	Alkyl
Ligand...VAL155	4.797	Pi-Alkyl
Ligand...MET94	5.009	Pi-Alkyl
Ligand...VAL155	4.467	Pi-Alkyl

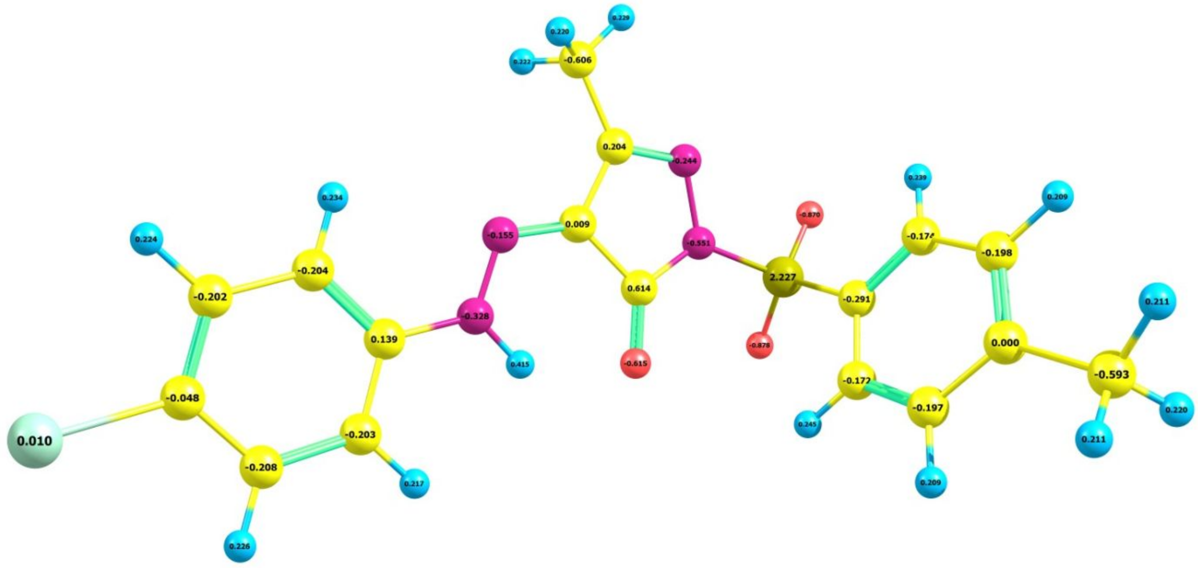

Figure S2: Atomic charges of PYRA-1

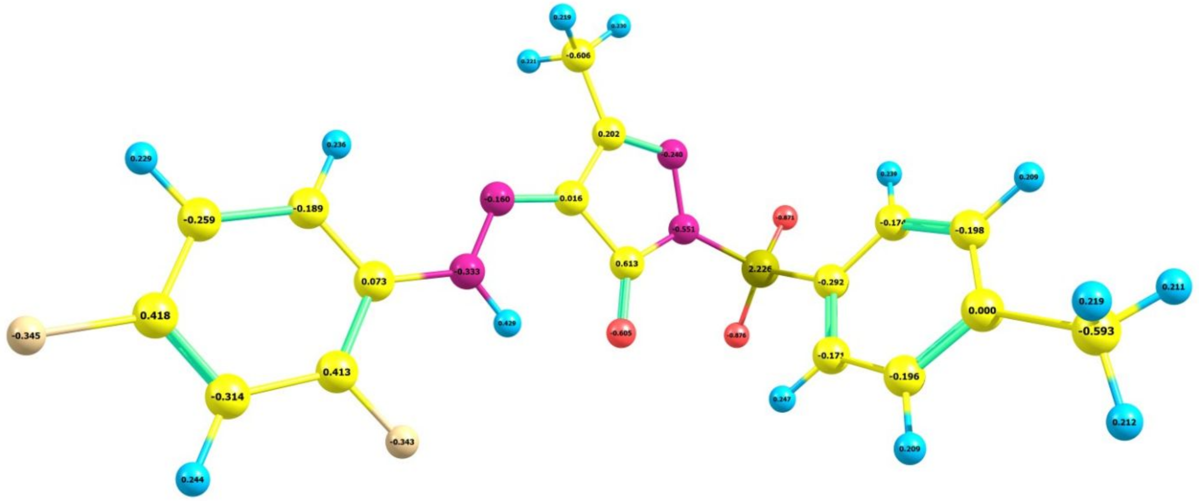

Figure S3: Atomic charges of PYRA-2

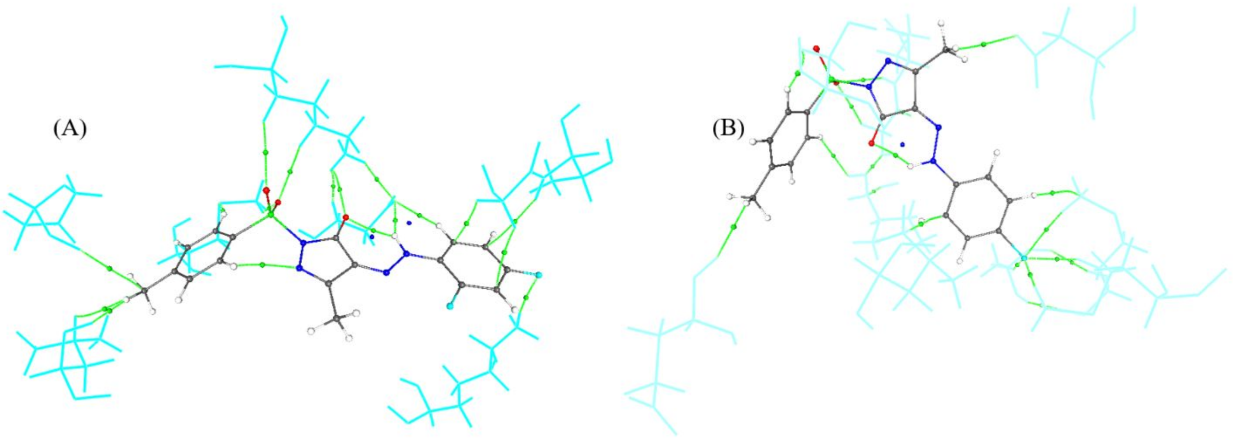

Figure S4: BCP map of (A) PYRA-1 and (B) PYRA-2 in the protein environment

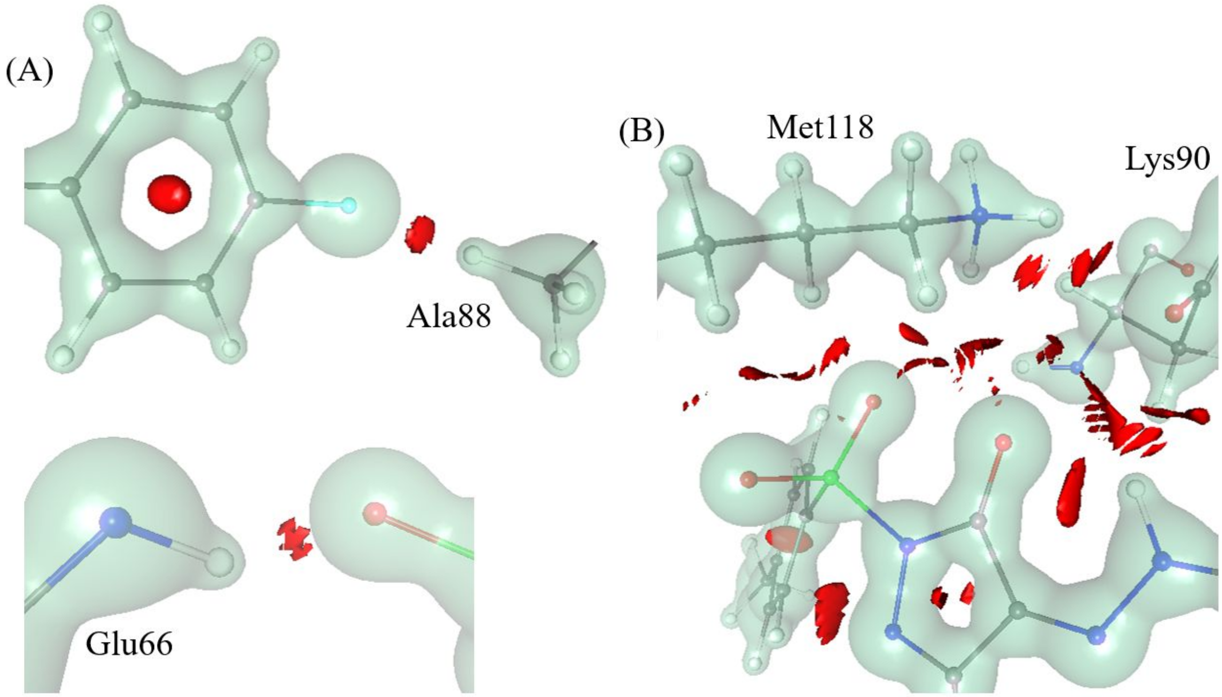

Figure S5: NCI isosurface map of protein-ligand complexes of (A) PYRA-1 and (B) PYRA-2 in the protein environment.

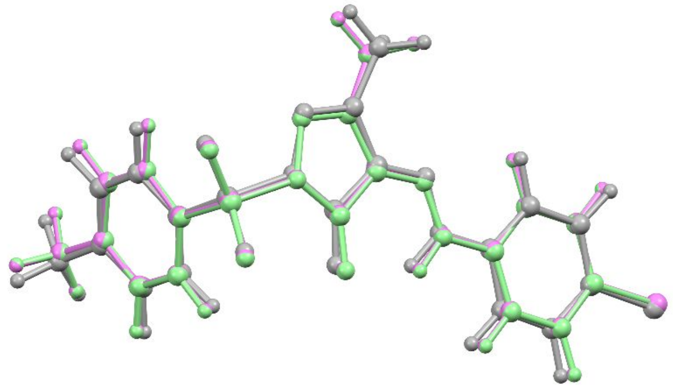

(a)

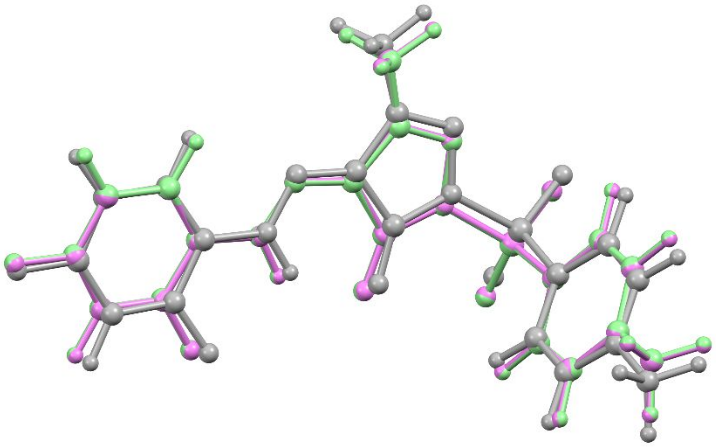

(b)

Figure S6: The superimposed view of gas phase (gray) (a) and solution phase (water: light green; DMSO: violet) (b).

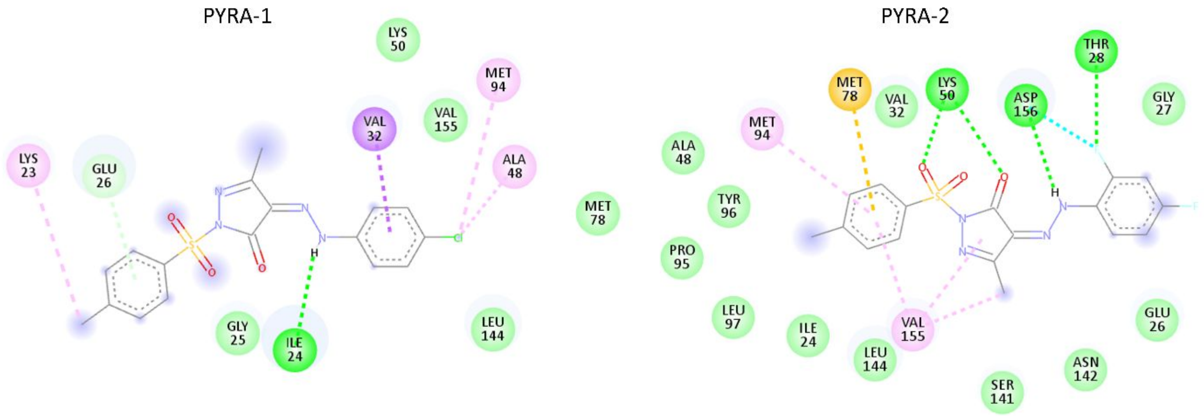

Figure S7: The 2D view of intermolecular interactions observed from 100ns of MD simulation.
